# Investigating Mitochondrial Viscosity in Ferroptosis‐Mediated Drug‐Induced Liver Injury using a Double‐Targeted Strategy

**DOI:** 10.1002/advs.202510425

**Published:** 2025-12-16

**Authors:** Yongqing Zhou, Yan Wang, Bing Huang, Hosoowi Lee, Mei Yan, Juyoung Yoon

**Affiliations:** ^1^ School of Chemistry and Chemical Engineering University of Jinan Jinan 250022 P. R. China; ^2^ Department of Chemistry and Nanoscience Ewha Womans University Seoul 03760 South Korea; ^3^ Graduate Program in Innovative Biomaterials Convergence Ewha Womans University Seoul 03760 South Korea

**Keywords:** double‐targeted strategy, ferroptosis‐mediated drug‐induced liver injury, mitochondrial viscosity, near‐infrared fluorescent probe, uncoupling protein 2

## Abstract

Investigating mitochondrial viscosity in ferroptosis‐mediated drug‐induced liver injury (DILI) is helpful for the reliable diagnosis and therapy of liver injury. Nevertheless, mitochondrial function and membrane potential may be impaired in the occurrence and development of DILI, so accurately monitoring viscosity changes remains a difficult task. Considering the presence of high expression of mitochondrial uncoupling protein 2 (UCP2) in liver injury, a new near‐infrared fluorescent probe (named FTZ‐2) is presented to investigate the tanglesome relationships between mitochondrial viscosity and ferroptosis‐mediated DILI by introducing a mitochondrial double‐targeted strategy that combined electrostatic reaction and probe–protein docking. The newly synthetized FTZ‐2 is highly selective to environmental viscosity in the presence of reactive species. Owing to its favorable cytotoxicity and mitochondrial localization characteristics, FTZ‐2 is used to monitor viscosity variations in ferroptosis cells and ferroptosis‐mediated DILI mice. The reduction of fluorescence signals indicated that ferrostatin‐1, glutathione, and N‐acetyl‐L‐cysteine can alleviate liver injury. Notably, the high expression of UCP2 is also discovered in the liver of ferroptosis mice and ferroptosis‐mediated DILI mice. Taken together, this work demonstrated a double‐targeted strategy for the early diagnosis and evaluation of liver injury through mitochondrial viscosity variations and contributed to the improvement of the therapeutic effect against liver injury.

## Graphical abstract

Utilizing a mitochondrial double‐targeted strategy, a novel near‐infrared fluorescent probe (FTZ‐2) is established for monitoring viscosity variations in ferroptosis‐mediated drug‐induced liver injury. In summary, this work provided a practical strategy for the early diagnosis and evaluation of liver injury.

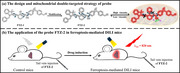

## 1 Introduction

Drug‐induced liver injury (DILI) is caused by immediate drug toxicity or complex metabolic reactions, which pose a major threat to human health.^[^ ^1^, ^2^ ^]^ With the incidence increasing, DILI has become a clinically common liver disease and a global public health problem.^[^ ^3^ ^]^ The occurrence and development of DILI are a complex process involving many factors. Drugs and their metabolites can covalently bind to cellular proteins and then trigger the immune response system, ultimately resulting in hepatotoxicity.^[^ ^4^, ^5^ ^]^ Reactive metabolites can cause the detoxification of endogenous reactive sulfur molecules, such as glutathione (GSH). In some cases, reactive sulfur species are severely depleted by excessive reactive metabolites, and then induce oxidative stress and mitochondrial damage, leading to liver injury and disorders.^[^ ^6^ ^]^ Presently, the clinical diagnosis of DILI relies on the analysis of the activity of blood enzymes, mainly including alanine aminotransferase and aspartate aminotransferase.^[^ ^7^, ^8^ ^]^ Unsatisfactorily, it is difficult to achieve early diagnosis of liver injury due to the unconspicuous early symptoms of DILI and the low sensitivity of enzyme detection in liver injury.

Recent studies have shown that liver diseases display various ferroptosis features, such as iron metabolism disorders and lipid peroxidation accumulation.^[^ ^9^, ^10^ ^]^ Moreover, ferroptosis regulation can intervene in the occurrence and progression of liver diseases.^[^ ^11^ ^]^ The mitochondria are not only the centers of energy generation but also the key sites for the production of reactive oxygen species (ROS).^[^ ^12^, ^13^, ^14^, ^15^ ^]^ As the main source of free iron ions, the mitochondria are highly susceptible to oxidative stress.^[^ ^16^ ^]^ As a catalyst, accumulation of iron ions promotes hydrogen peroxide to produce hydroxyl radical (·OH) with strong oxidizing ability through the Fenton reaction and then gives rise to the release of other ROS.^[^ ^17^, ^18^ ^]^ Moreover, the overexpression of ROS damages intracellular reactive macromolecules, such as proteins, lipids, and DNA, accompanied by intracellular microenvironment changes, such as viscosity.^[^ ^19^, ^20^ ^]^ On the contrary, variations in the mitochondrial microenvironment accelerate excessive aggregation of iron, which aggravates mitochondrial damage and cell death.^[^ ^21^ ^]^ To date, mitochondrial viscosity can be used as an important indicator for providing a vital diagnostic platform for the monitoring onset and process of ferroptosis‐mediated DILI and the evaluation of new therapeutic drugs of DILI.

Mitochondrial uncoupling proteins (UCPs) are a group of conserved family proteins located on the inner mitochondrial membrane.^[^ ^22^ ^]^ They can permeate the mitochondrial proton gradient and release energy in the form of heat. Among them, mitochondrial uncoupling protein 2 (UCP2), widely distribute in the liver and immune cells, regulates energy balance and antioxidant defense.^[^ ^23^ ^]^ Currently, some reports have discovered that UCP2 is involved in numerous liver diseases, such as hepatitis, liver fibrosis, and liver cancer.^[^ ^24^, ^25^ ^]^ Moreover, the abnormal high expression of UCP2 in non‐alcoholic fatty liver disease leads to mitochondrial dysfunction under pathological conditions.^[^ ^26^ ^]^ Impressively, we speculated that there was an abnormal overexpression in the onset and development of DILI. Hence, preparing a reliable tool for monitoring UCP2 levels may be used for the early diagnosis of DILI, including ferroptosis‐mediated DILI.

Small‐molecule fluorescent probes have become valuable tools to detect some biomarkers in living systems, particularly microenvironmental parameters (e.g., viscosity), owing to high specificity, adjustable structure, and versatility.^[^ ^27^, ^28^, ^29^, ^30^, ^31^, ^32^, ^33^, ^34^, ^35^, ^36^ ^]^ Numerous viscosity‐responsive fluorescent probes have been synthesized and applied to the analysis of molecular events in biological systems.^[^ ^37^, ^38^, ^39^, ^40^, ^41^, ^42^ ^]^ Depending on the molecular structure, positively charged molecular probes are generally used to explore mitochondrial function, activity, and spatial distribution in living cells. Nevertheless, the mitochondrial targeting ability of this type of fluorescent probe may be influenced by other elements, such as mitochondrial membrane potential (MMP), and ion concentrations.^[^ ^43^, ^44^ ^]^ In some cases, partial probes may diffuse or detach from the mitochondria, affecting the accuracy and reliability of the measurement results to some extent. On this basis, the development of improved mitochondrial targeting molecular fluorescent probes for the dependable detection of viscosity variations is critical in disease diagnosis.

Molecular docking helps with the understanding of the interactions between molecules with docking substances through inherent driving forces (such as hydrogen bonds, Van der Waals forces, and *π*–*π* interactions) or spatial associative conformation.^[^ ^45^ ^]^ Up to now, this docking method is universally employed in the construction and screening of enzyme‐responsive fluorescent probes, which visibly improve targeting performance and detection accuracy. Significatively, compared with positive charge targeting mitochondria, this method showed more fascinating properties, such as greater bonding ability and decreasing the interference of mitochondrial membrane potential. For instance, employing a molecular docking strategy, Lin's group developed a new molecular probe for evaluating the therapeutic effect of ferroptosis on myocardial ischemia–reperfusion injury.^[^ ^46^ ^]^ Unfortunately, this probe had not been applied in living organisms. Based on this typical study, the molecular docking method can be widely applied to other diagnosis areas, such as ferroptosis‐mediated DILI.

**Scheme 1:**
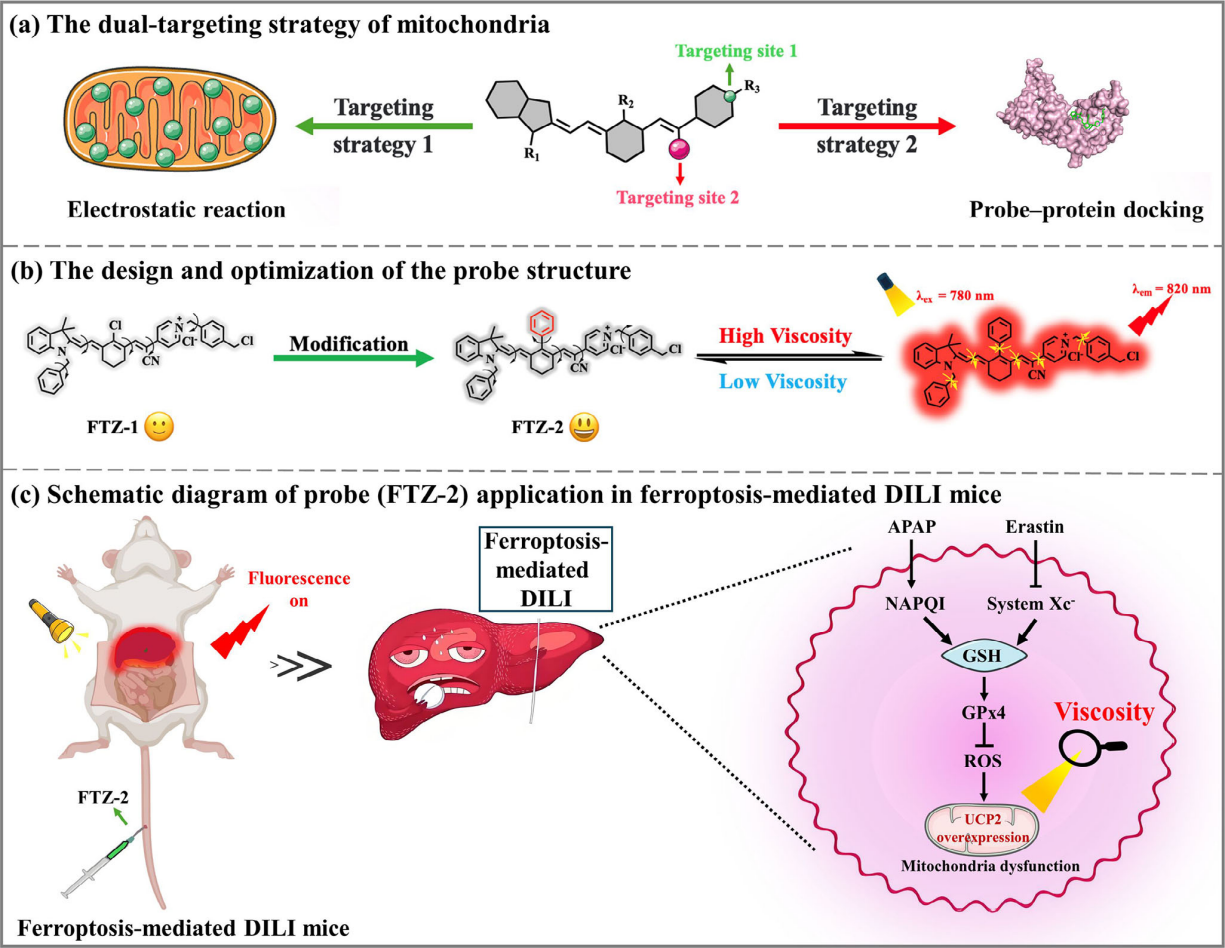
a) The mitochondria dual‐targeting strategy of the probe. b) The design and optimization of the probe structure. c) Schematic diagram of probe (FTZ‐2) application in ferroptosis‐mediated DILI mice.

Therein, by combining electrostatic absorption and probe‐protein docking principle, we engineered two novel viscosity‐responsive near‐infrared (NIR) small‐molecule fluorescent probes (FTZ‐1 and FTZ‐2). Thereinto, the FTZ‐2 was composed of three components: the single bond in a new NIR fluorophore as a motor, a positive charge that could specifically target the mitochondria (electrostatic forces), and a cyano group of FTZ‐2 that could form a hydrogen bond with a tyrosine residue of UCP2 (probe‐protein docking), further enhancing the targeting ability (**Scheme** **1**). FTZ‐2 showed more fascinating viscosity responsiveness in in vitro experiments than FTZ‐1, because the Cl atom was replaced by a benzene ring. In the colocalization experiment, under the action of the positively charged unit and the cyano group, FTZ‐2 primarily targeted the mitochondrial region, achieving accurate positioning. Molecular docking results indicated the interaction between the cyano group of FTZ‐2 and the lysine residue (TYR 156) of UCP2 by forming a noncovalent bond (hydrogen bond). Compared with the previous probes (Table S1, Supporting Information), the probe FTZ‐2 displayed several advantages, such as high mitochondrial targeting ability and NIR emission. Intracellular viscosity changes were consecutively investigated upon treatment with acetaminophen (APAP) or erastin and so on. In view of the advantages of NIR emission (*λ*_em_ = 820 nm) in high‐viscosity media, FTZ‐2 was employed to investigate the complicated relationships between mitochondrial viscosity and ferroptosis‐mediated DILI.

## 2 Results and Discussion

### 2.1 Probe Design and Characterization

The probe (FTZ‐1) structure consisted of merocyanine, 2‐pyridin‐4‐ylacetonitrile, and 1,4‐bis (chloromethyl) benzene. The environmental viscosity was determined by limiting the rotation of the single bond in the probe structure. To improve the response of the fluorescent probe to viscosity, the Cl atom of probe FTZ‐1 was replaced by a benzene ring to obtain probe FTZ‐2, owing to the addition of a larger motor. The probes with donor (D)‐*π*‐acceptor (A) structures showed rotor‐like features, which were similar to some typical examples. Moreover, the positively charged unit and the cyano moiety were served as the targetable groups for the mitochondria and UCP2, respectively. Additionally, the characteristics of the probes and other compounds were verified through high‐resolution mass spectrometry and nuclear magnetic resonance. The detailed synthetic routes of the two probes were presented in Schemes S1 and S2 (Supporting Information).

### 2.2 Optical Properties of the Probes

**Figure 1:**
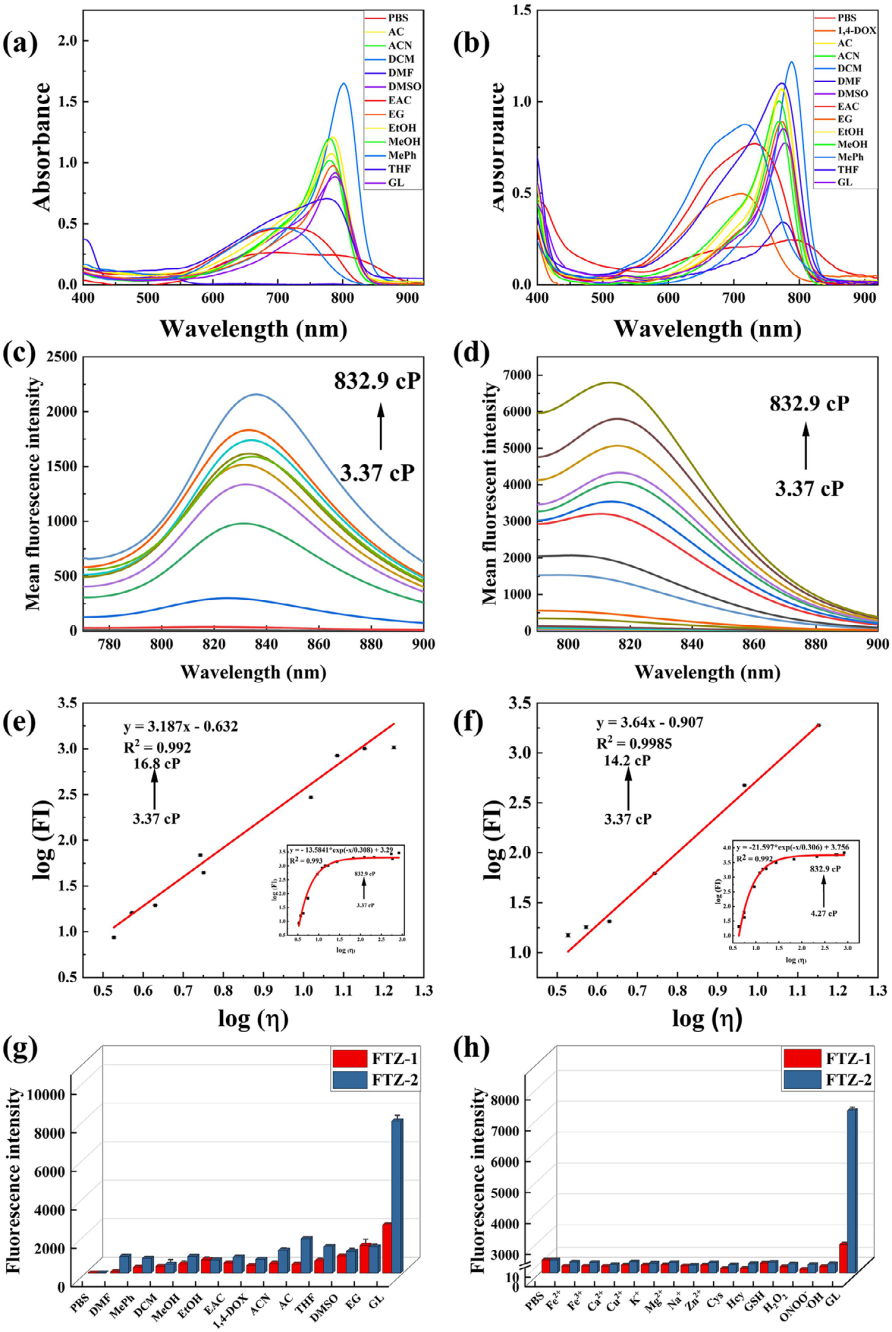
a,b) The absorption spectra of FTZ‐1 and FTZ‐2 in different solvents. c,d) The fluorescence emission spectra of FTZ‐1 and FTZ‐2 in solutions of different viscosities. e,f) Linear fitting of log (η) and log (FI_830_) and log (FI_820_) for FTZ‐1 and FTZ‐2. g,h) Selectivity tests of FTZ‐1 and FTZ‐2. *λ*_ex (FTZ‐1)_ = 750 nm, *λ*_em (FTZ‐1)_ = 830 nm. *λ*_ex (FTZ‐2)_ = 780 nm, *λ*_em (FTZ‐2)_ = 820 nm.

Initially, the absorption and fluorescence spectra of two probes (FTZ‐1 and FTZ‐2) were measured in solvents of different polarities and viscosities. The absorption peaks of the probes were principally concentrated in the NIR region (**Figure** **1**a,b). In detail, the apparent absorption peaks at 750 nm (FTZ‐1) and 780 nm (FTZ‐2) were displayed in high‐viscosity solvents (glycerol). Two fluorescent probes fluoresced weakly in several low‐viscosity solvents, such as PBS and methanol. However, significant fluorescence enhancements (FTZ‐1 at 830 nm, FTZ‐2 at 820 nm) were clearly appeared in high viscosity solvents. Surprisingly, compared with the probes in PBS, the fluorescence intensities of the two probes in glycerol substantially increased by 553 and 841 times. These phenomena indicated that two NIR fluorescence probes were more responsive to viscosity than to polarity. The fluorescence intensities of the two probes were successively measured in PBS–glycerol mixtures with various proportions of glycerol to further demonstrate that the two probes responded to environmental viscosity changes. The viscosity value of the mixed solution was expressed by η. As expected, the fluorescence intensities of the probes steadily increased as the glycerol ratio in the mixed systems increased. Moreover, log (FI) enhanced linearly with log (η) (Figure 1c–f). Thus, high correlation coefficients of R^2^ = 0.992 for FTZ‐1 and R^2^ = 0.999 for FTZ‐2 were obtained, confirming that the two probes were highly sensitive to environmental viscosity variations. In addition, the fluorescence quantum yields also gradually increased as the viscosity of the mixed solution increased. Whereafter, the selectivity experiments of the two probes were evaluated against potential interferents. As shown in Figure 1g,h, the two probes both emitted remarkable fluorescence signals in glycerol. However, the weak fluorescence was observed in solutions containing various metal ion and reactive species. These findings demonstrated that the two developed probes could be used to monitor viscosity in complex biological systems. The stabilities of the two probes were thoroughly tested in different pH solutions. Their fluorescence intensities were basically unchanged in the media with a pH range of 4.0–9.0 (Figure S1a,c, Supporting Information). Therefore, they were not influenced by environmental pH. Moreover, two fluorescent probes exhibited high and stable fluorescent signals after continuous irradiation for 1.0 h in glycerol (Figure S1b,d, Supporting Information), certifying that two fluorescent probes possessed satisfactory photostability and monitored viscosity over time. Collectively, two fluorescent probes could be applied in monitoring viscosity fluctuations in complex systems.

### 2.3 Cytotoxicity and Targeting Capacity of the Probes

**Figure 2:**
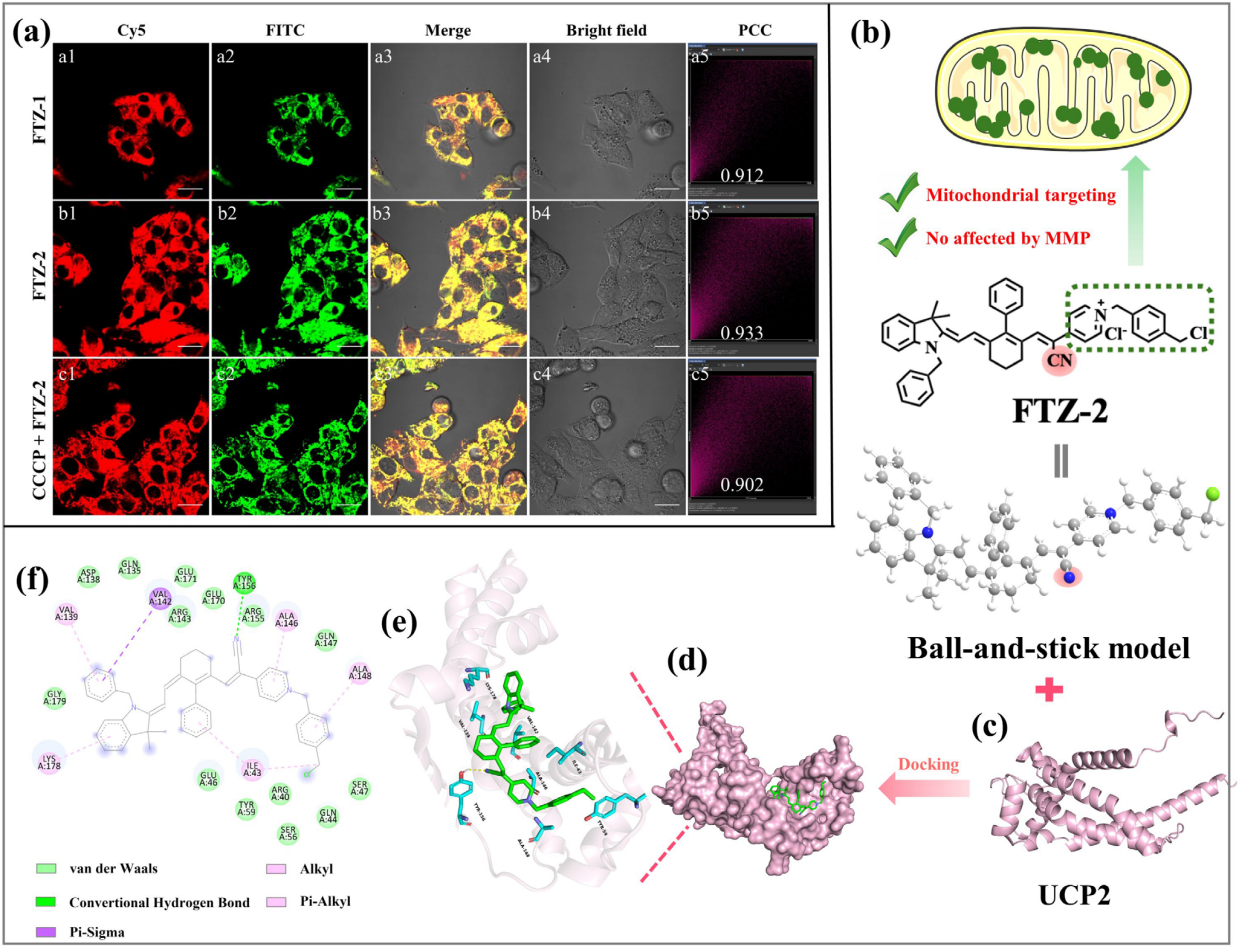
a) Mitochondrial colocalization imaging of FTZ‐1 (a1–a4) and FTZ‐2 (b1–b4 and c1–c4) in HepG2 cells in the absence and presence of CCCP (10 µm, 2 h). b) Schematic of the double‐targeting strategy of FTZ‐2. c) Structure of UCF2. d) Overview of the conformational arrangement of UCP2 and FTZ‐2. e) Neighboring residues of UCP2 and FTZ‐2. h) Diagram of UCF2 docking with FTZ‐2. Scale bar: 20 µm. Cy5 channel: FTZ‐1 (8.0 µM, λ_ex_ = 640 nm, λ_em_ = 663 ‐ 738 nm), FITC channel: Mito‐Tracker Green (150 nM, λ_ex_ = 488 nm, λ_em_ = 500‐550 nm).

The cytotoxicities of the two probes were assessed using 5‐dimethylthiazol‐2‐yl‐2,5‐diphenyltetrazolium bromide (MTT) prior to cellular experiments. As shown in Figure S2 (Supporting Information), cell viability remained above 80% in both HeLa cells and HepG2 cells at high experimental concentrations (25.0 µm), demonstrating that the two probes possessed low cytotoxicity. The characters of the probes in detecting intracellular viscosity changes were then investigated in HeLa and HepG2 cells. Previous reports indicated that positively charged probes usually showed a satisfying mitochondrial targeting ability because of electrostatic interactions. To verify the mitochondrial targeting ability, co‐localization imaging of two probes was performed using a commercial mitochondrial tracker dye (Mito‐Tracker Green). The targeting specificity was evaluated in living cells through Pearson Correlation Coefficient (PCC) analysis of the fluorescence overlap between red channel and green channel. As demonstrated in **Figure** **2**a1–a4,b1–b4, the fluorescence of these two channels perfectly coincided, with a high PCC (FTZ‐1 for 0.912, FTZ‐2 for 0.933), indicating that the probes were mainly concentrated in mitochondria. Then, the ability of the FTZ‐1 and FTZ‐2 to target into other organelles was also explored. The FTZ‐2 was almost absent from other organelles (Figures S3 and S4, Supporting Information), such as lipid droplets, nucleus, lysosomes, endoplasmic reticulum, Golgi apparatus. Subsequently, the targeting ability of the FTZ‐2 was also investigated when the mitochondrial membrane potential was reduced. Carbonyl cyanide 3‐chlorophenylhydrazone (CCCP), a chemical reagent that reduces mitochondrial membrane potential, was chose to study potential membrane potential‐dependent targeting behavior. As expected, FTZ‐2 also demonstrated excellent mitochondrial localization (PCC = 0.902) in depolarized mitochondria (Figure 2c1–c4). To verify the positioning effect of the probe under higher polarization states, the cells was treated with high concentrations of CCCP (20 µm). Surprisingly, the FTZ‐2 still demonstrated excellent targeting toward mitochondria in the presence of high concentrations of CCCP (Figure S5, Supporting Information, PCC = 0.891 in HepG2 cells, Figure S6, Supporting Information, PCC = 0.914 in HeLa cells). Similarly, the FTZ‐1 also displayed decent mitochondrial targeting ability (Figure S7, Supporting Information, PCC = 912)in HeLa cells. Therein, these results preliminarily confirmed that FTZ‐2 possessed strong mitochondrial targeting ability and was not affected by membrane potential changes. To further confirm the locating capability of the FTZ‐2, the detailed molecular information of the binding sites and surrounding amino acid residues of UCP2 were obtained by molecular docking method, since UCP2 exists in the mitochondrial inner membrane. The homologous molecular docking results manifested that the interaction between cyano group of FTZ‐2 and a tyrosine residue (TYR 156) of UCP2 via forming a non‐covalent bond (hydrogen bond) to partially embed into the protein cavity (Figure 2b–d). FTZ‐2 also exhibited hydrophobic interactions with VAL142, ILE43, VAL139, LYS178, ALA146, and ALA148 in UCF2 (Figure 2e,f). Therefore, FTZ‐2 primarily collected into UCP2 through probe‐protein docking, further enhancing the targetable capability. For a more direct understanding of the docking content, the molecular configuration and interactions of the FTZ‐2 were represented. Thus, the mitochondria targeting accuracy of the FTZ‐2 was improved with the help of the positive charge and cyano group via the mitochondrial double‐targeted strategy.

### 2.4 Assessment of Mitochondrial Oxidative Stress

Next, the detection capabilities of the two probes (FTZ‐1 and FTZ‐2) were examined to evaluate the mitochondrial oxidative stress status. Lipopolysaccharide (LPS) induces mitochondrial oxidative stress because it not only generates superfluous ROS but also causes a noticeable viscosity fluctuation.^[^ ^47^ ^]^ Nystatin (Nys) is a common intracellular ionophore, which induce mitochondrial structural and internal viscosity changes, due to disruption of ion homeostasis.^[^ ^48^ ^]^ The detection capability of FTZ‐2 was first tested. As shown in Figure S8 (Supporting Information), LPS‐/Nys‐/APAP‐stimulated cells emitted dazzling fluorescence signals, testifying that the mitochondrial viscosity increased significantly under mitochondrial oxidative stress. The fluorescence intensity was distinctly reduced upon treatment with N‐acetyl‐L‐cysteine (NAC, a ROS scavenger, relieving oxidative stress). The similar phenomenon was observed using FTZ‐1 (Figure S9, Supporting Information) in HeLa cells under the same experimental conditions. The evidence strongly supported that the probes had a sufficient capacity to monitor mitochondrial viscosity variations.

### 2.5 Monitoring of Mitochondrial Viscosity in APAP‐Induced DILI Cells

**Figure 3:**
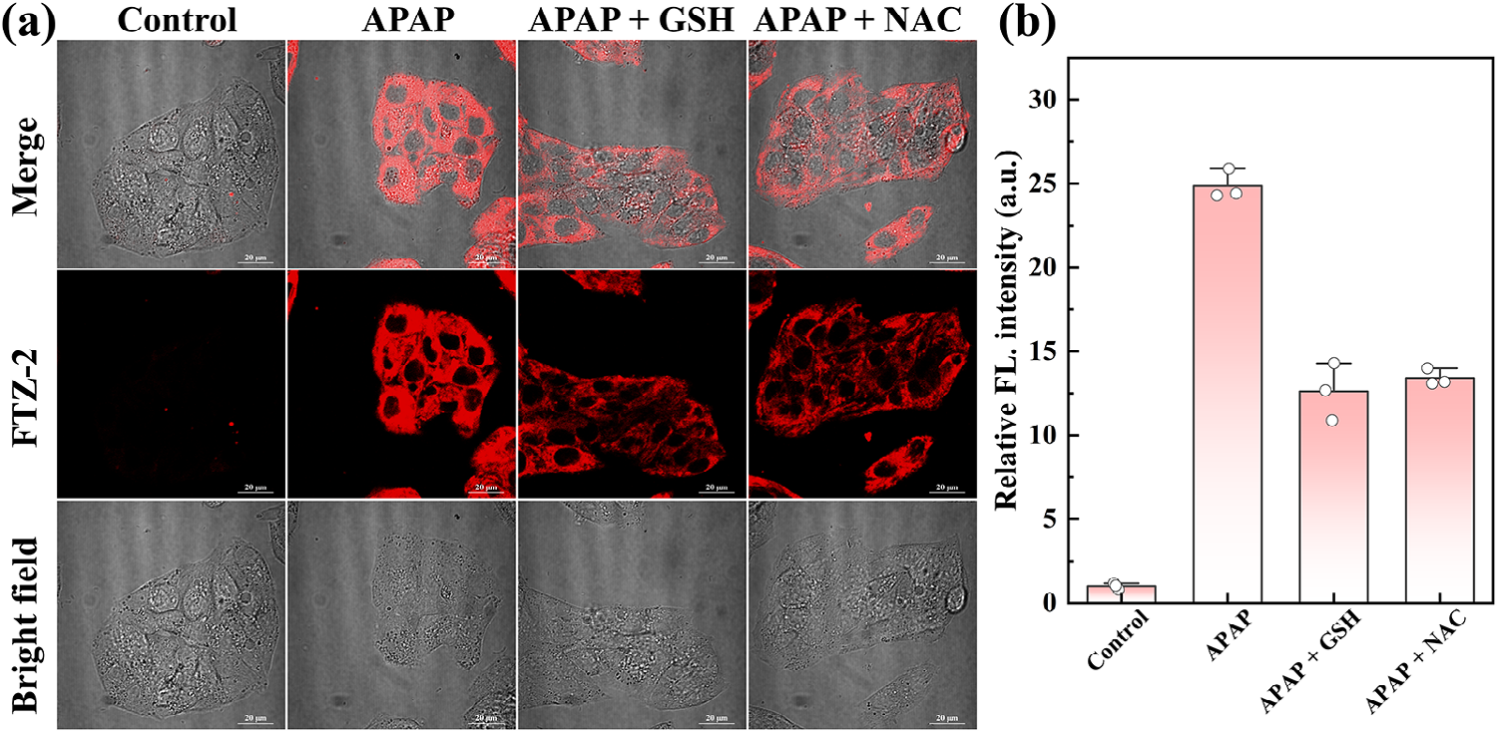
a) Cellular fluorescence imaging of mitochondrial viscosity in DILI HepG2 cells. b) Relative fluorescent intensity of each group.

The liver is the main organ involved in drug metabolism.^[^ ^49^ ^]^ In some situations, certain drugs are metabolized and converted into hepatotoxic substances when they enter the liver. These substances consume amounts of glutathione (GSH) and then decrease the reducing capacity of GSH‐dependent GPx4, thereby trigger redox imbalances in liver cells, ultimately leading to the accumulation of lipid peroxidation and liver cell damage.^[^ ^50^ ^]^ Before visual tools are used to investigate the roles of ferroptosis in DILI, mitochondrial viscosity as reliable indicators for evaluating the severity of DILI should be determined. For instance, acetaminophen (APAP), a common antipyretic and analgesic drug, induces hepatic damage through metabolic activation into the reactive toxic intermediate N‐acetyl‐p‐benzoquinone imine (NAPQI), which depletes intracellular GSH, initiating a cascade of oxidative stress and mitochondrial dysfunction, along with mitochondrial viscosity fluctuations, ultimately resulted in liver damage.^[^ ^51^, ^52^ ^]^ APAP‐incubated cells exhibited intense red fluorescence signals compared with the control cells. Therefore, mitochondrial viscosity increased significantly in APAP‐induced DILI cells (**Figure** **3**). Some reactive oxygen scavengers (such as GSH and NAC) can effectively reduce the degree of DILI. Similar to the experimental results, upon treatment with NAC or GSH, the stronger red fluorescence was suppressed, indicating that mitochondrial damage was readily alleviated, and mitochondrial viscosity was distinctly reduced. Therefore, these combined results indicated that FTZ‐2 might be an effective tool for the visual monitoring of mitochondrial viscosity in DILI cells.

### 2.6 Monitoring of Mitochondrial Viscosity in Ferroptosis Cells

Ferroptosis, an iron‐dependent programmed cell death, is characterized by lipid ROS accumulation.^[^ ^53^, ^54^ ^]^ Iron, as a catalyst, has the ability to produce ·OH from hydrogen peroxide through the Fenton reaction. The superfluous iron promotes a burst of intracellular ROS, leading to intracellular oxidative stress and lipid peroxidation,^[^ ^55^, ^56^ ^]^ accompanied by internal microenvironmental variations. Mitochondria are the main ROS producing sites in the cell, and are prone to oxidative stress in some cases. On this basis, mitochondrial viscosity changes were roundly investigated in an Fe^2^⁺‐induced ferroptosis cell model. The significant fluorescence intensity was observed in Fe^2^⁺‐induced ferroptosis cells compared with that in the control controls, indicating an increased mitochondrial viscosity. The treatment with deferiprone (DFP, an iron chelator) resulted in fluorescent intensity reduction (Figure S10, Supporting Information). These findings demonstrated that iron participated in ferroptosis and the mitochondrial viscosity increased overtly during ferroptosis.

**Figure 4:**
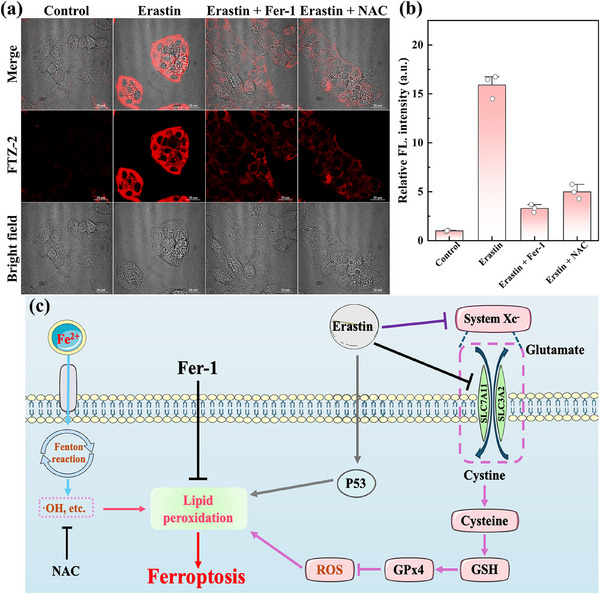
a) Confocal fluorescent imaging of erastin‐induced HepG2 cells. b) Relative fluorescent intensity of each group. c) Schematic of ferroptosis. *λ*_ex_ = 640 nm, *λ*_em_ = 663–738 nm. Scale bar: 20 µm.

Presently, erastin, an inducer of ferroptosis, triggers intracellular ferroptosis by reducing the efficiency of system Xc^−^ (**Figure** **4**). And then, it decreases glutathione synthesis, resulting in the accumulation of deleterious free radicals, such as ·OH, which further oxidizes and destroys some biomolecules, together with viscosity change.^[^ ^57^ ^]^ P53, a vital transcription factor for SLC7A11 (a crucial transporter responsible for mediating cysteine uptake), can facilitate the extent of erastin‐induced ferroptosis by inhibiting the SLC7A11 expression.^[^ ^58^, ^59^ ^]^ The mitochondrial viscosity changes were explored in erastin‐induced ferroptosis. The results indicated that the fluorescence intensity in the erastin‐induced ferroptosis cells was higher than that of other groups. Moreover, as shown in Figure S11 (Supporting Information), the intracellular fluorescence intensity elevated sharply with increasing doses of erastin, displaying a positive correlation, and suggested that oxidative stress was also gradually increasing in HeLa cells. The results confirmed that mitochondrial viscosity increased markedly in ferroptosis cells. Ferrostatin‐1 (Fer‐1, a lipophilic antioxidant and potent ferroptosis inhibitor), can inhibit ferroptosis.^[^ ^60^ ^]^ The fluorescence intensities in the inhibition groups (Fer‐1, NAC) were significantly reduced compared with those in the ferroptosis group. Therefore, mitochondrial viscosity also decreased in the presence of several inhibitions. The coincident phenomenon was observed in HeLa cells (Figure S12, Supporting Information). Therefore, these experimental findings confirmed that the mitochondrial viscosity of ferroptosis cells increased significantly.

In addition, isoniazid (INH), an antituberculosis drug, can consume intracellular GSH and induce ferroptosis^[^ ^61^ ^]^ (Figure S13a, Supporting Information). To reveal the mitochondrial viscosity changes during ferroptosis upon exposure to INH, we primitively observed the fluorescent signals of FTZ‐2 in living cells treated with different concentrations of INH. The experimental results demonstrated that the fluorescence intensities in the Fer‐1/GSH/NAC‐stimulated cells were obviously lower than those in the ferroptosis cells, but they remained higher than those of the control cells (Figure S13b,c, Supporting Information). Notably, the intracellular fluorescence intensity presented an upward trend with the increase in INH levels (Figure S14, Supporting Information). Thus, the incremental fluorescence intensity directly reflected a corresponding increasement in mitochondrial viscosity, further confirming that a positive correlation between INH‐induced ferroptosis and mitochondrial viscosity.

### 2.7 Monitoring of Mitochondrial Viscosity in Ferroptosis‐Mediated DILI Cells

**Figure 5:**
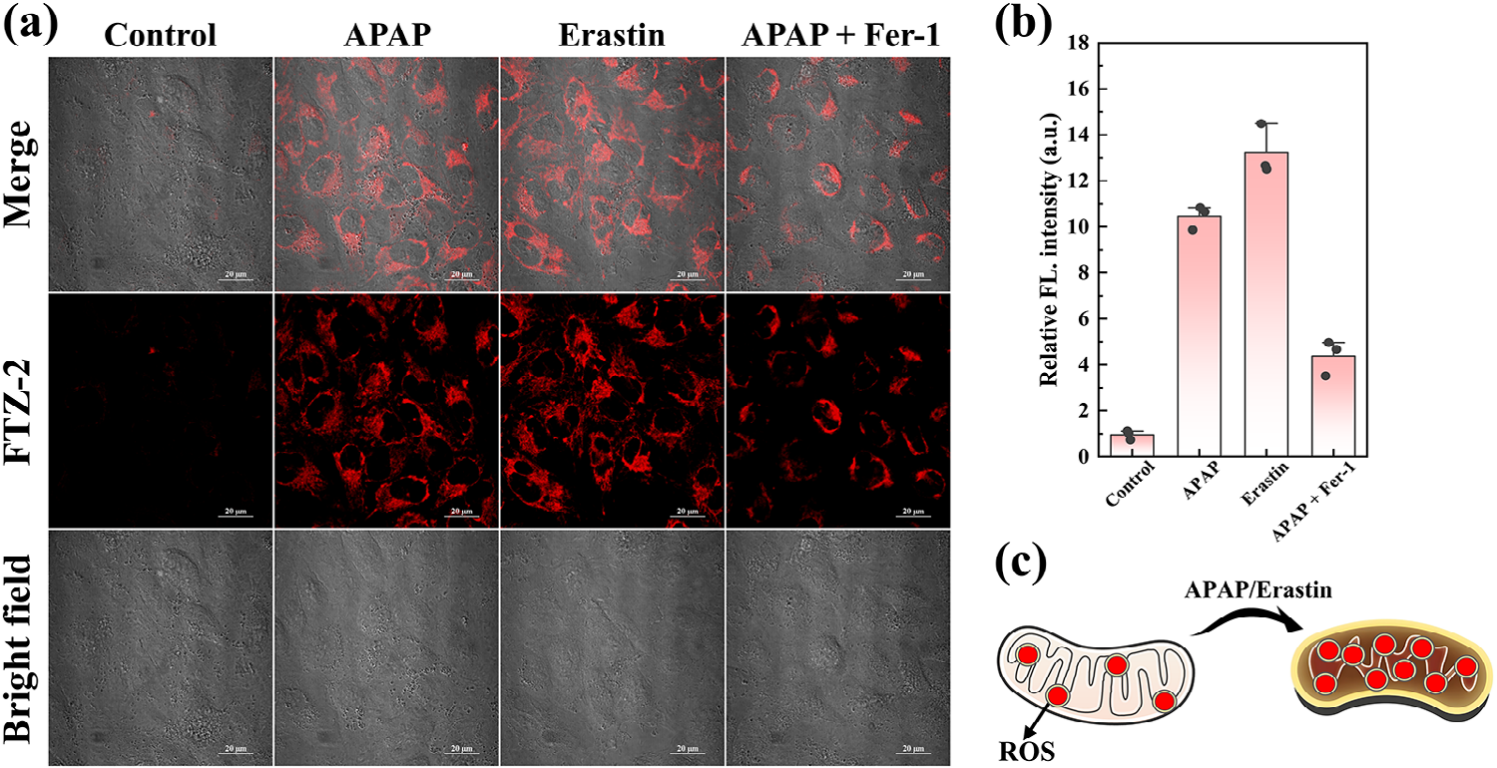
a) Fluorescent imaging of the mitochondrial viscosity in ferroptosis‐mediated DILI in HepG2 cells. b) Relative fluorescent intensity of each group. c) Schematic of DILI cells. *λ*_ex_ = 640 nm, *λ*_em_ = 663–738 nm. Scale bar: 20 µm.

Recent studies have shown that various liver diseases show different ferroptosis degrees, such as lipid peroxidation accumulation and abnormal changes in the microenvironment.^[^ ^62^ ^]^ As such, a comprehensive understanding ferroptosis in the process of DILI is essential for the reliable diagnosis and effective treatment of liver diseases. To investigate the ability of FTZ‐2 and detect mitochondrial viscosity fluctuations in DILI, we established the corresponding cell models by using APAP and erastin. As shown in **Figure** **5**a,b,c, compared with the control group, the APAP‐induced and erastin‐induced cells both send out stronger fluorescence, suggesting that mitochondrial viscosity increased significantly in these cells. Especially, upon exposure to Fer‐1, strong fluorescence of APAP‐induced cells was efficaciously suppressed, indicating that inhibiting the degree of ferroptosis could alleviate DILI. Therefore, the experimental results verified that FTZ‐2 could be used to monitor ferroptosis‐mediated DILI via mitochondrial viscosity imaging.

### 2.8 Monitoring of Mitochondrial Viscosity in Ferroptosis‐Mediated DILI Mice

**Figure 6:**
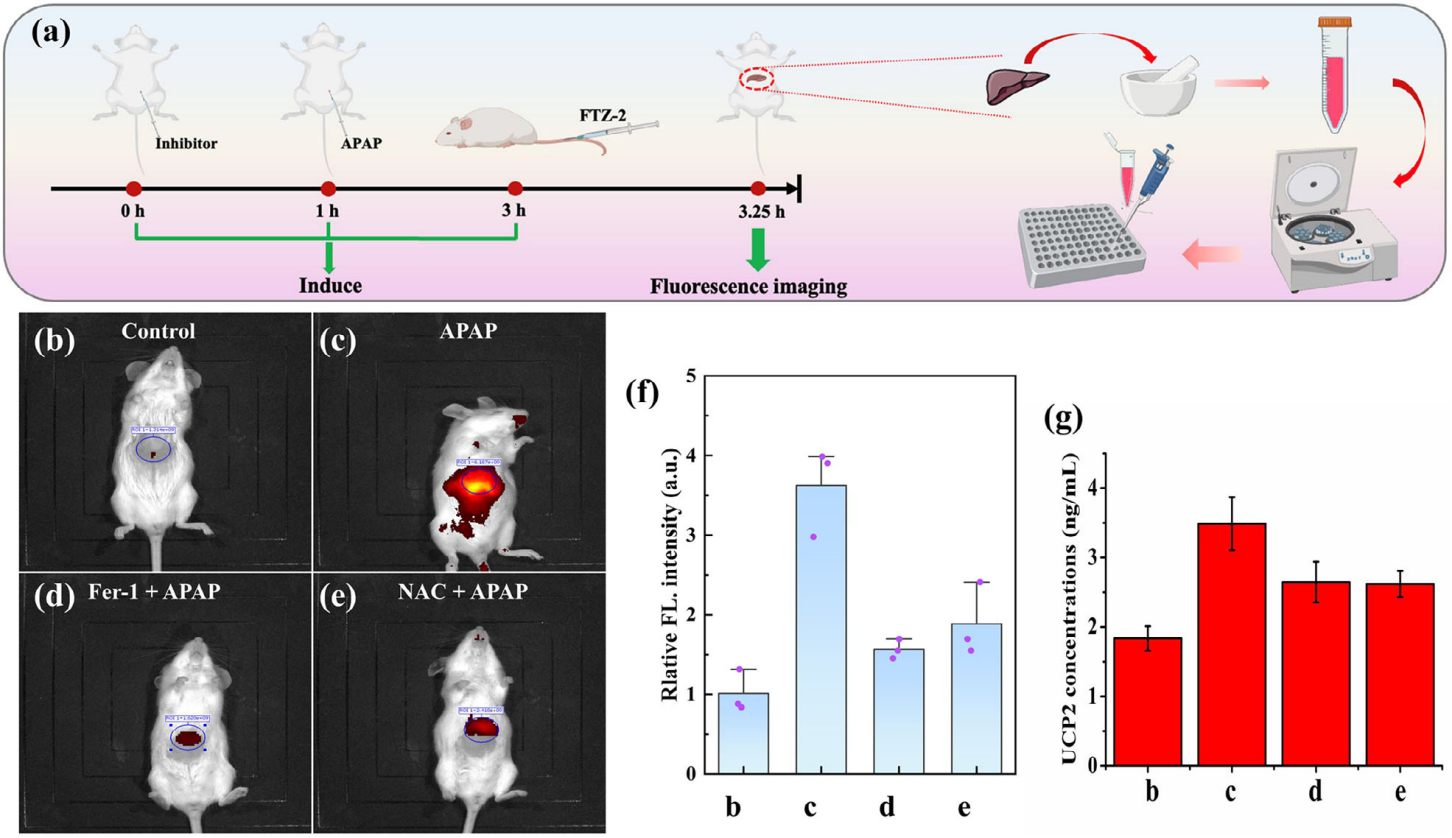
a) Diagram of the process for in mice, organ imaging, and protein content determination. b–e) In vivo imaging of each mouse group. f) Relative fluorescent intensity of each group. g) Histogram of the conversion concentration of UCP2 in the liver homogenate of each mouse group. *λ*_ex_ = 740 nm, *λ*_em_ = 790 nm.

Encouraged by the favorable results of FTZ‐2 in cells, we used it to expand its imaging capabilities to verify viscosity in DILI mice and ferroptosis‐mediated DILI mice. The KM mice were randomly assigned to four groups: the control group, the APAP group, the NAC+APAP group, and the Fer‐1+APAP group. As shown in **Figure** **6**a–f, in comparison to the other three groups (control group, NAC+APAP group and the Fer‐1+APAP group), the DILI group exhibited the strongest fluorescence intensity, indicating that the viscosity of fluorescence sites of DILI group augmented obviously. Noteworthily, the NAC+APAP and Fer‐1+APAP groups displayed a modest reduction in fluorescence intensity, yet still remained higher than that of control mice, suggesting that the viscosity was reduced in two groups and ferroptosis accelerated the development of DILI. These findings also indicated that NAC and Fer‐1 elicited a certain therapeutic effect on ferroptosis‐mediated DILI. The mouse organs were harvested for fluorescence imaging to further demonstrate the location of fluorescence production. The corresponding experimental results were consistent with the in vivo fluorescence imaging data (Figure S15, Supporting Information). Detailedly, compared with the control mice, the liver of the APAP‐treated mice emitted a stronger fluorescence. However, after the addition of the NAC and Fer‐1, the intense near‐infrared fluorescence was suppressed. This phenomenon indicated that the viscosity within the liver of the DILI mice was significantly increased. Further, Fer‐1 and NAC both possessed a certain effect in alleviating liver damage. Moreover, the obvious fluorescence was mainly concentrated in the mouse liver. Subsequently, given the overexpression of UCP2 in DILI, the UCP2 levels were measured in ferroptosis‐mediated DILI mice. Figure 6g certified that the content of UCP2 in liver tissue of APAP‐treated mice was obviously enhanced, while the UCP2 levels of mouse liver in NAC+APAP‐and Fer‐1+APAP‐treated groups were slightly increased. Furthermore, the pathological features of these mouse livers were assessed via hematoxylin and eosin (H&E) staining (Figure S16, Supporting Information). Histopathological evaluation revealed that hepatocytes in the control group maintained normal morphology, such as well‐defined nuclear membranes, absence of necrosis, and no evidence of vacuolar degeneration. In contrast, the APAP‐treated group exhibited severe hepatic injury characterized by disorganized cellular architecture, dilated and congested hepatic sinusoids, structural disintegration, pronounced eosinophilic staining, and extensive necrotic lesions. While the liver cells in Fer‐1 and NAC‐treated groups were eosinophilic, but there was no obvious damage, indicating that both Fer‐1 and NAC alleviated DILI to a certain extent. Therein, ferroptosis was indeed involved in the occurrence and development of DILI.

**Figure 7:**
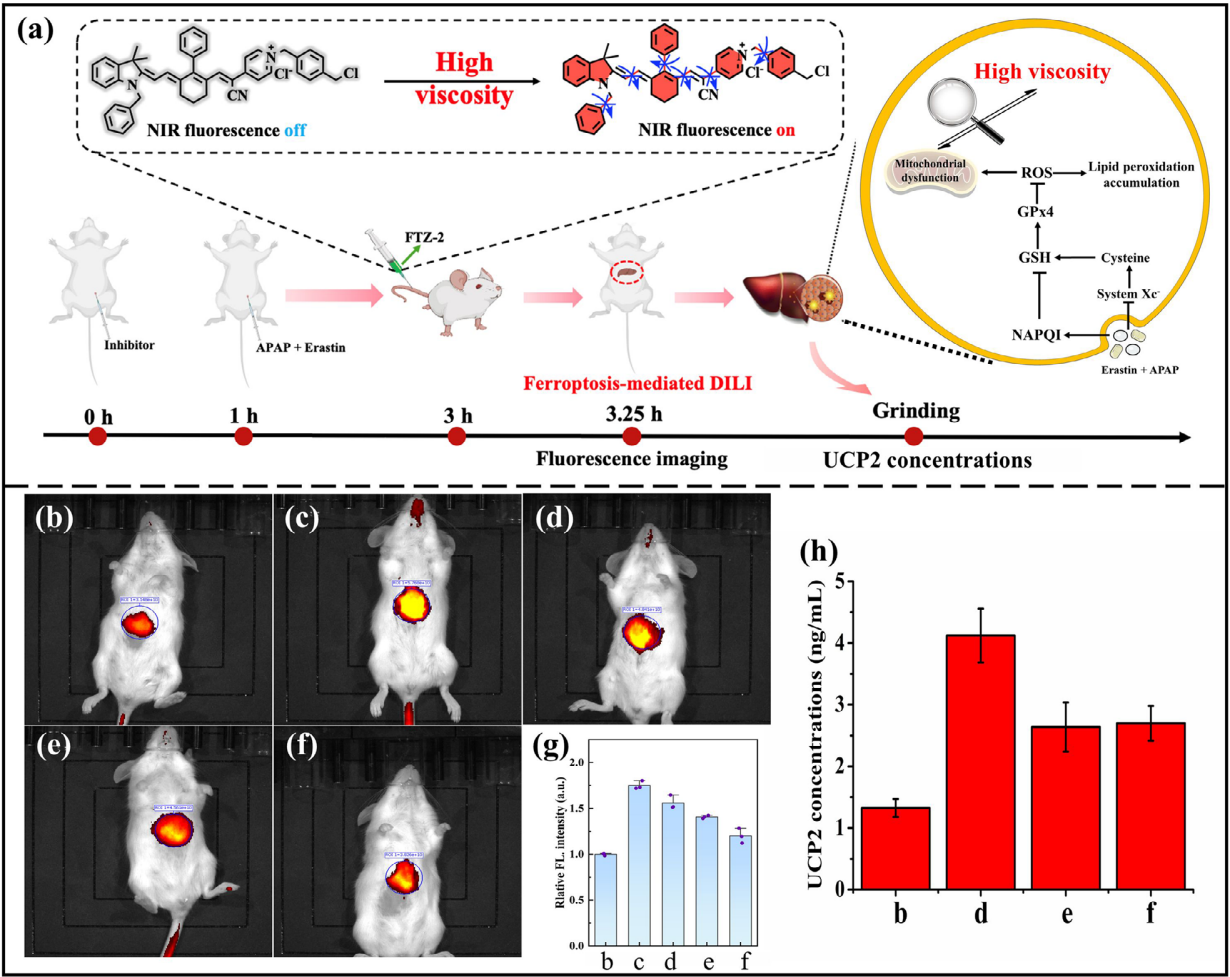
a) Schematic diagram of ferroptosis‐mediated DILI. In vivo fluorescence imaging of b) Control group, c) APAP+erastin group, d) erastin group, e) Fer‐1+ APAP+erastin group, f) Fer‐1+erastin group. g) Relative fluorescent intensity of each group. h) Histogram of the concentration of UCP2 in the liver homogenate of each mouse group. *λ*_ex_ = 740 nm, *λ*_em_ = 790 nm.

Then, to further investigate the viscosity variations in ferroptosis mice and ferroptosis‐mediated DILI of mice, KM mice were randomly assigned to five groups. As shown in **Figure** **7**a–g, In comparison to control mice, erastin‐induced mice exhibited brighter fluorescence, illustrating that the viscosity was elevated in erastin‐induced mice. Notably, the strongest fluorescence signals were observed in APAP+erastin‐treated mice, indicating that the mouse viscosity was the highest. With the treatment of NAC or Fer‐1, fluorescence was suppressed. Moreover, the results of mouse organ imaging were consistent with those of mouse live imaging (Figure S17, Supporting Information). Hence, the test results confirmed that ferroptosis plays an important role in DILI. Moreover, the overexpression of UCP2 in the APAP+Erastin‐treated mice was measured to verify fluorescent imaging findings (Figure 7h). Additionally, H&E staining (Figure S18, Supporting Information) verified whether it was simultaneously induced by APAP and Erastin or erastin alone; the mouse liver showed varying degrees of edema and vacuolar degeneration. Nevertheless, the liver of APAP‐ and erastin‐treated mice displayed extensive vascular congestion. The Fer‐1‐treated group showed significant relief from the liver damage. These results demonstrated that Fer‐1 could relieve and treat DILI by inhibiting ferroptosis.

## 3 Conclusion 

In summary, we cleverly constructed a novel viscosity‐sensitive NIR fluorescent probe via introducing a double‐targeted strategy that integrated electrostatic interaction and probe‐protein docking. In vitro test, FTZ‐2 displayed higher responsive traits, such as high selectivity. Moreover, the FTZ‐2 mainly targeted into the mitochondria under the action of a positively charged unit and a cyanide group, allowing us to dynamically detect mitochondrial viscosity changes in ferroptosis and ferroptosis‐mediated DILI cells. Furthermore, employing FTZ‐2, the higher viscosity was observed in ferroptosis‐mediated DILI mice. Importantly, we evaluated the therapeutic effects of several other reagents (NAC, GSH, and Fer‐1) on liver injury at different viscosities. We found that Fer‐1 could alleviate DILI to a certain extent and ferroptosis was indeed involved in the occurrence and development of DILI. Collectively, the novel viscosity‐sensitive probe constructed with a mitochondrial dual‐targeting strategy could serve as a novel tool for the reliable diagnosis and treatment of ferroptosis‐mediated DILI.

## Conflict of Interest

The authors declare no conflict of interest.

## Ethical Statement

KM mice (weighting 24–26 g, aged 4 weeks, female) were purchased from Ji’ nan Pengyue laboratory Animal Breeding CO., Ltd. All animal care and experimental protocols complied with the Animal Management Rules of the Ministry of Health of the People's Republic of China and were approved by the Animal Care Committee of Shandong Normal University. (Ethics Approval Number: AEECSDNU2025080).

## Supporting information



Supporting Information

## Data Availability

The data that support the findings of this study are available in the supplementary material of this article.
