# Supplementary material for: Investigating Mitochondrial Viscosity in Ferroptosis‐Mediated Drug‐Induced Liver Injury using a Double‐Targeted Strategy
Source: Adv Sci (Weinh). 2025 Dec 16;13(11):e10425. doi: 10.1002/advs.202510425 (PMC12931212; doi:10.1002/advs.202510425)
Supplement: Supplementary file 1 — Supporting Information [file ADVS-13-e10425-s001.docx]

**Supporting Information**

Investigating Mitochondrial Viscosity in Ferroptosis-mediated Drug-induced Liver Injury Using a Double-targeted Strategy

Yongqing Zhou,*^,#[a]^ Yan Wang,^#[a]^ Bing Huang,^[a]^ Hosoowi Lee,^[b]^ Mei Yan,*^[a]^ Juyoung Yoon*^[b,c]^

1. School of Chemistry and Chemical Engineering, University of Jinan, Jinan 250022, People’s Republic of China
2. Department of Chemistry and Nanoscience, Ewha Womans University, Seoul 03760, Korea
3. Graduate Program in Innovative Biomaterials Convergence, Ewha Womans University, Seoul 03760, Korea

^#^Yongqing Zhou and Yan Wang contributed equally.

*To whom correspondence should be addressed.

E-mail: [chm_zhouyq@ujn.edu.cn](mailto:chm_zhouyq@ujn.edu.cn) , [chm_yanm@126.com](mailto:chm_yanm@126.com). jyoon@ewha.ac.kr

Contents

[1. Materials and Methods 3](#_Toc215010331)

[1.1 Materials and instruments 3](#_Toc215010332)

[1.2 Synthesis and characterization of FTZ-1 and FTZ-2 3](#_Toc215010333)

[1.3 In vitro experimental methods 5](#_Toc215010334)

[1.4 Molecular docking calculation method 6](#_Toc215010335)

[2. Cell Imaging 6](#_Toc215010336)

[2.1 Cell culture and cytotoxicity assays 6](#_Toc215010337)

[2.2 Co-localization experiment 7](#_Toc215010338)

[2.3 Induction and imaging methods of cellular inflammation and drug-induced liver injury models 7](#_Toc215010339)

[2.4 Induction and imaging methods of endogenous and exogenous ferroptosis models in cells 8](#_Toc215010340)

[3. Living and Organ Imaging 8](#_Toc215010341)

[4. Synthetic route of FTZ-1 and FTZ-2 9](#_Toc215010342)

[5. Supplementary Figures 12](#_Toc215010343)

[6. ^1^H NMR, ^13^C NMR and HRMS Spectra 20](#_Toc215010344)

# 1. Materials and Methods

## 1.1 Materials and instruments

Unless otherwise stated, all reagents were purchased from commercial suppliers and used without further purification. Twice-distilled water was used throughout all experiments. AVANCE III 400 MHz and AVANCE III HD 600 MHz were used for nuclear magnetic resonance spectroscopy testing. High resolution mass spectrometric (HRMS) tests were measured on Orbitrap IQ-X Mass spectrometer. Absorption spectra was obtained on DH-2000-BAL spectrometers. Photoluminescent spectra was recorded with HITACHI F4600 fluorescence spectropho-tometer with a 1 cm standard quartz cell. The fluorescence imaging of cells was performed with Nikon A1 MP confocal microscopy. The living imaging of mice was performed with IVIS Lumina XRMS Series III. The pH measurements were carried out on a Mettler-Toledo Delta 320 pH meter.

## 1.2 Synthesis and characterization of FTZ-1 and FTZ-2

**Synthesis of compound 4:** Compound 3 (403 mg, 1 mmol) and 4-Pyridylacetonitrile (141.6 mg, 1.2 mmol) were dissolved in EtOH (10 mL) and Piperidine (100 μL) was added into the mixture solution. The mixture solution was stirred at 78 °C for 4 h. Silica gel column purification was performed by using PE: EA = 2:1 to obtain compound 4 as a purple solid. ^1^H NMR (400 MHz, DMSO-d_6_) δ 8.59 (d, J = 5.3 Hz, 2H), 8.11 (s, 1H), 7.63 (d, J = 12.5 Hz, 1H), 7.51 (d, J = 5.2 Hz, 2H), 7.40 – 7.12 (m, 8H), 6.94 (t, J = 6.8 Hz, 2H), 5.08 (s, 2H), 2.89 (t, J = 6.0 Hz, 2H), 2.40 (t, J = 6.4 Hz, 2H), 1.75 – 1.67 (m, 2H), 1.61 (s, 6H). ^13^C NMR (150 MHz, DMSO-d_6_) δ 162.07, 150.89, 144.39, 143.09, 142.60, 142.50, 139.16, 136.91, 131.76, 129.19, 128.50, 127.68, 127.00, 126.15, 124.14, 122.45, 121.59, 119.71, 118.51, 108.31, 103.54, 95.09, 46.63, 45.67, 28.79, 28.44, 25.79, 21.42. HRMS (ESI, m/z): calcd. 504.2208, found: 504.2202 for [M+H]^+^.

**Synthesis of FTZ-1:** Compound 4 (503 mg, 1 mmol), α,α'-Dichloro-p-xylene (348 g, 2 mmol) and K_2_CO_3_ (79 mg, 0.5 mmol) were dissolved in acetonitrile (10 mL). The mixture solution was stirred at 85 °C for 17 h. Silica gel column purification was performed by using DCM: MeOH = 10:1 to obtain FTZ-1 as a green solid. ^1^H NMR (400 MHz, DMSO-d_6_) δ 8.85 (d, J = 6.7 Hz, 2H), 8.25 (s, 1H), 8.00 (dd, J = 15.0, 10.0 Hz, 3H), 7.60 – 7.43 (m, 6H), 7.41 – 7.10 (m, 10H), 5.72 (s, 2H), 5.35 (s, 2H), 2.95 (d, J = 6.2 Hz, 2H), 1.77 (s, 2H), 1.34 – 1.13 (m, 8H). ^13^C NMR (150 MHz, DMSO-d_6_) δ 168.72, 152.08, 148.46, 144.40, 143.91, 143.53, 141.52, 140.33, 139.66, 139.07, 136.13, 135.39, 130.08, 129.37, 129.27, 128.03, 127.04, 125.53, 125.49, 123.95, 122.86, 121.27, 118.65, 110.47, 98.92, 94.76, 70.25, 61.55, 55.40, 48.31, 29.48, 28.34, 25.83, 21.22. HRMS (ESI, m/z): calcd. 642.2438, found: 642.2435 for [M-Cl]^+^.

**Synthesis of compound 5:** Compound 3 (161.2 mg, 0.4 mmol), Phenylboronic acid (195.1 mg, 1.6 mmol), and K_3_PO_4_ (987.2 mg, 4.7 mmol), Pd (PPh_3_)_4_ (190 mg, 0.16 mmol) were dissolved in DMF/H_2_O (25 mL, v/v = 5:1). The mixture solution was stirred at 120 °C for 24 h. After cooling to room temperature, the mixture was dissolved in ethyl acetate (50 mL) and washed with brine (3×100 mL). The organic layer was dried over Na_2_SO_4_, and concentrated under reduced pressure. Aluminum oxide column purification was performed by using PE: EA = 20:1 to obtain compound 5 as an orange solid. ^1^H NMR (400 MHz, DMSO-d_6_) δ 9.11 (s, 1H), 7.47 (d, J = 6.7 Hz, 3H), 7.36 – 7.07 (m, 10H), 6.84 (dt, J = 7.3, 3.4 Hz, 2H), 6.40 (d, J = 12.8 Hz, 1H), 4.99 (s, 2H), 2.36 (dt, J = 24.9, 6.1 Hz, 4H), 1.69 (t, J = 6.2 Hz, 2H), 1.08 (s, 6H). ^13^C NMR (150 MHz, DMSO-d_6_) δ 191.37, 160.72, 158.12, 144.54, 138.86, 137.45, 137.07, 134.01, 130.89, 130.31, 129.15, 128.70, 128.40, 128.32, 128.05, 127.59, 126.95, 122.37, 121.01, 107.72, 94.21, 45.88, 45.54, 28.09, 25.08, 22.56, 21.34. HRMS (ESI, m/z): calcd. 446.2486, found: 446.2481 for [M+H]^+^.

**Synthesis of compound 6:** Compound 5 (445 mg, 1 mmol) and 4-Pyridylacetonitrile (141.6 mg, 1.2 mmol) were dissolved in EtOH (10 mL) and Piperidine (100 μL) was added into the mixture solution. The mixture solution was stirred at 78 °C for 4 h. Silica gel column purification was performed by using PE: EA = 2:1 to obtain compound 6 as a purple solid. ^1^H NMR (400 MHz, DMSO-d_6_) δ 8.46 – 8.40 (m, 2H), 7.56 – 7.45 (m, 3H), 7.36 – 7.28 (m, 3H), 7.28 – 7.06 (m, 9H), 7.04 – 6.95 (m, 2H), 6.88 – 6.81 (m, 2H), 6.31 (d, J = 12.9 Hz, 1H), 5.01 (s, 2H), 2.90 (t, J = 6.2 Hz, 2H), 2.40 (d, J = 6.1 Hz, 2H), 1.89 – 1.75 (m, 2H), 1.07 (s, 6H). ^13^C NMR (150 MHz, DMSO-d_6_) δ 160.74, 150.74, 145.39, 144.47, 143.58, 138.98, 137.05, 134.85, 130.24, 129.18, 129.13, 128.70, 128.41, 128.21, 128.11, 127.63, 127.13, 126.97, 122.41, 121.17, 118.86, 107.89, 99.48, 45.99, 45.58, 28.40, 28.03, 27.04, 24.46. HRMS (ESI, m/z): calcd. 546.2911, found: 546.2905 for [M+H]^+^.

**Synthesis of FTZ-2:** Compound 6 (545 mg, 1 mmol), α,α'-Dichloro-p-xylene (348 g, 2 mmol) and K_2_CO_3_ (79 mg, 0.5 mmol) were dissolved in acetonitrile (10 mL). The mixture solution was stirred at 85 °C for 17 hours. Silica gel column purification was performed by using DCM: MeOH = 10:1 to obtain FTZ-2 as a green solid. ^1^H NMR (400 MHz, DMSO-d_6_) δ 8.63 (d, J = 7.0 Hz, 2H), 7.57 – 7.53 (m, 2H), 7.51 – 7.39 (m, 6H), 7.39 – 7.02 (m, 16H), 6.85 – 6.79 (m, 1H), 5.56 (s, 2H), 5.28 (s, 2H), 3.01 – 2.89 (m, 2H), 2.47 (d, J = 5.4 Hz, 2H), 1.92 – 1.81 (m, 2H), 1.11 (s, 6H). ^13^C NMR (150 MHz, DMSO-d_6_) δ 168.39, 161.94, 152.04, 146.59, 144.27, 143.47, 143.38, 140.19, 138.99, 138.50, 136.14, 135.32, 130.76, 130.24, 130.02, 129.35, 129.29, 129.03, 128.81, 128.73, 128.42, 128.00, 127.25, 127.03, 123.76, 122.80, 119.33, 119.13, 110.25, 98.70, 49.07, 47.75, 45.97, 28.33, 27.86, 26.68, 24.35, 21.58. HRMS (ESI, m/z): calcd. 684.3141, found: 684.3138 for [M-Cl]^+^.

## 1.3 In vitro experimental methods

The solution of FTZ-1 and FTZ-2 (1 mM) was prepared in DMSO, storing at -20 °C. The final concentration of probes in all vitro experiments was 10 μM. The testing solutions were all treated by ultrasound for 10 min to eliminate bubbles.

The selectivity test of FTZ-1 and FTZ-2 was carried out in this series of solutions. A series of interference species were added to solutions, Na^+^ (1 mM), Ca^2+^ (500 μM), Cu^2+^ (10 μM), Fe^2+^ (10 μM), Fe^3+^ (10 μM), K^+^ (1 mM), Mg^2+^ (500 μM), Zn^2+^ (10 μM), Cl^-^ (1 mM), GSH (1 mM), Cys (10 μM), Hcy (10 μM), ONOO^-^ (10 μM), H_2_O_2_ (1 mM), ·OH (10 μM). For all the measurements, the excitation wavelength of FTZ-1 was 750 nm, the excitation wavelength of FTZ-2 was 780 nm. The excitation slit widths, emission slit widths and excitation voltage was 10.0 nm, 10.0 nm, and 700 V, respectively.

The fluorescent quantity yield of FTZ-2 in different GL volumes of PBS/GL systems was calculated. The cresol violet was regarded as a reference. Among them, cresol violet was dissolved in methanol, while FTZ-2 was dissolved in DMSO and the testing concentration of FTZ-2 was 10 μM. The specific experimental data was shown in Table 1. As the proportion of GL increasing, the fluorescence quantum yield continued to increase. Finally, in the pure GL system, the fluorescence quantum yield reached 0.203%.

The fluorescent quantity yield of FTZ-2 in solutions of different proportions of PBS/GL.


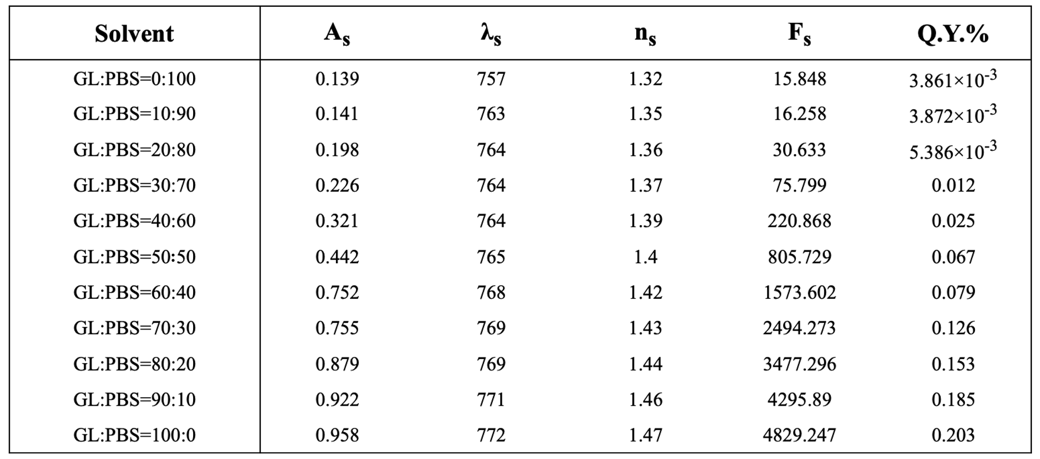


## 1.4 Molecular docking calculation method

The protein structure was retrieved from the RCSB PDB database (uniprotID: A6I6P3). Molecular docking was subsequently conducted using Autodock vina, while the 3D structure of the small molecule was generated with Chem 3D. The final data was provided from Scientific Compass www.shiyanjia.com.

# 2. Cell Imaging

## 2.1 Cell culture and cytotoxicity assays

HeLa cells and HepG2 cells were utilized in this work. HeLa cells and HepG2 cells were cultured in Dulbecco’s Modified Eagle Medium media (DMEM, Basal-Media) supplemented with 10% heat-inactivated fetal bovine serum (FBS, Biosharp) at 37 °C and 5% CO_2_.

The cytotoxicity of FTZ-1 and FTZ-2 to HeLa cells and HepG2 cells was studied by standard microculture tetrazolium (MTT) assays. 1 × 10^6^ cells/mL cells were seeded in 96-well plates and then incubated with various concentrations of FTZ-1 and FTZ-2 (0 - 25 μM) for 12 h. After that, 10 μL of MTT (0.5 mg / mL) was added to each well and incubated for another 4 h. Finally, the media was discharged, and 100 μL DMSO was added to dissolve the formazan crystals. The plate was shaken for about 10 min, and each well was analyzed by the microplate reader and detected at the absorbance of 490 nm. The cell viability (%) = (OD_sample_ – OD_blank_) / (OD_control_ – OD_blank_) × 100%. (OD_sample_, OD_control_, and OD_blank_ denote the cells incubated with various concentrations of probes, the cells without probes, and the wells containing only the culture media, respectively). HeLa cells and HepG2 cells showed more than 80% cell viability below 25 μM of FTZ-1 and FTZ-2, which suggested it has low cytotoxicity to living cells at a specific concentration. Therefore, the concentration of 8 μM of FTZ-1 and FTZ-2 was chosen for the subsequent cellular experiments.

## 2.2 Co-localization experiment

HepG2 cells and HeLa cells were used in the co-localization experiments, and all commercial dyes were purchased from suppliers. Among them, Mito-Tracker Green (150 nM, 15 min, λ_ex_ = 488 nm, λ_em_ = 500-550 nm) and FTZ-2 (8 μM, 15 min, λ_ex_ = 640 nm, λ_em_ = 663-738 nm) were incubated for imaging. Mito-Tracker Green (150 nM, 15 min, λ_ex_ = 488 nm, λ_em_ = 500-550 nm) and FTZ-1 (8 μM, 15 min, λ_ex_ = 640 nm, λ_em_ = 663-738 nm) were incubated for imaging. Lyso-Tracker Green (50 nM, 15 min, λ_ex_ = 488 nm, λ_em_ = 500-550 nm) was incubated with cells and then FTZ-2 (8 μM, 15 min, λ_ex_ = 640 nm, λ_em_ = 663-738 nm) was added. BODIPY (50 nM, 15 min, λ_ex_ = 488 nm, λ_em_ = 500-550 nm) and then FTZ-2 (8 μM, 15 min, λ_ex_ = 640 nm, λ_em_ = 663-738 nm) was added. CCCP (10/20 μM, 20 μM) was firstly incubated with HeLa cells for 2 h to depolarize mitochondria and then incubated with Mito-Tracker Green (150 nM, 15 min, λ_ex_ = 488 nm, λ_em_ = 500-550 nm) and FTZ-2 (8 μM, 15 min, λ_ex_ = 488 nm, λ_em_ = 500-550 nm) for cell imaging. All co-localization experiments were repeated three times.

## 2.3 Induction and imaging methods of cellular inflammation

Nystatin (Nys, 10 μM) or lipopolysaccharide (LPS, 5 μg/mL) was added to the inoculum with cells incubated for 30 min before adding probes (8 μM, 15 min), acetaminophen (APAP, 100 μM, 300 μM, 500 μM) was added to the inoculum with cells and incubated for 2 h before adding probes (8 μM, 15 min). N-acetyl-L-cysteine (NAC, 10 μM) was added after the successful induction of cell liver injury model by APAP. Each experiment was repeated three times. Wash the cells three times with PBS before imaging. λ_ex_ = 640 nm, λ_em_ = 663-738 nm.

## 2.4 Induction and imaging methods of ferroptosis model cells

Fe^2+^ (500 μM) incubated with HeLa cells for 6 h to induce cells exogenous ferroptosis, and then incubated with FTZ-2 (8 μM, 15 min) for cell imaging. DPF (500 μM, 2 h) was added into exogenous ferroptosis cells and then incubated with FTZ-2 (8 μM, 15 min) for cell imaging.

Eratin (1 μM, 3 μM, 5 μM) and isoniazid (INH, 100 μM, 300 μM, 500 μM) incubated with cells to induce endogenous ferroptosis, and then incubated with FTZ-2 (8 μM, 15 min) for cell imaging. Fer-1 (10 μM, 2 h) was firstly incubated with endogenous ferroptosis of cells and then incubated with FTZ-2 (8 μM, 15 min) for cell imaging. GSH (1 mM, 2 h) was firstly incubated with endogenous ferroptosis of cells and then incubated with FTZ-2 (8 μM, 15 min) for cell imaging. NAC (10 μM, 2 h) was firstly incubated with endogenous ferroptosis of cells and then incubated with FTZ-2 (8 μM, 15 min) for cell imaging.

Each experiment was repeated three times. Wash the cells three times with PBS before imaging. λ_ex_ = 640 nm, λ_em_ = 663 - 738 nm.

# 3. Mice and Mouse Organ Imaging

KM mice (weighting 24 - 26 g, aged 4 weeks, female) were purchased from Ji’ nan Pengyue laboratory Animal Breeding CO., Ltd. All animal care and experimental protocols complied with the Animal Management Rules of the Ministry of Health of the People’s Republic of China and were approved by the Animal Care Committee of Shandong Normal University. (Ethics Approval Number: AEECSDNU2025080). Before in vivo imaging, all mice were depilated in the abdomen to ensure the leakage of the liver for imaging purposes. In living experiments, control group mice were intraperitoneally injected with an equal amount of saline. The DILI mice model was induced and constructed by intraperitoneal injection of APAP (400 mg/kg, 2 h), while the NAC treated group mice model was constructed by intraperitoneal injection of NAC (400 mg/kg, 1 h) before intraperitoneal injection of APAP (400 mg/kg, 2 h), the Fer-1 treated group mice model was constructed by intraperitoneal injection Fer-1 (5 μM, 1 h) before intraperitoneal injection of APAP (400 mg/kg, 2 h). In addition, in the second group of live experiments, APAP (400 mg/kg, 2 h) + Erastin (10 μM, 2 h), Erastin (10 μM, 2 h), Fer-1 (5 μM, 1 h) + APAP (400 mg/kg, 2 h) + Erastin (10 μM, 2 h), Fer-1 (5 μM, 1 h) + Erastin (10 μM, 2 h) were constructed by intraperitoneally injection, and FTZ-2 (100 μM, 100 μL, 15 min) was injected through tail vein injection. After the in vivo imaging, the important tissues heart, liver, spleen, lung, and kidney of each mouse were dissected for organ imaging. Repeat each experiment three times to ensure the accuracy of the results. λ_ex_ = 740 nm, λ_em_ = 790 nm. The H&E staining, pathological analysis was supplied by Shandong Yuxiang Biotechnology Co., Ltd and UCP2 content determination of mice liver was supplied by Shanghai Yuanju Biotechnology Co., Ltd.

# 4. Synthetic route of FTZ-1 and FTZ-2


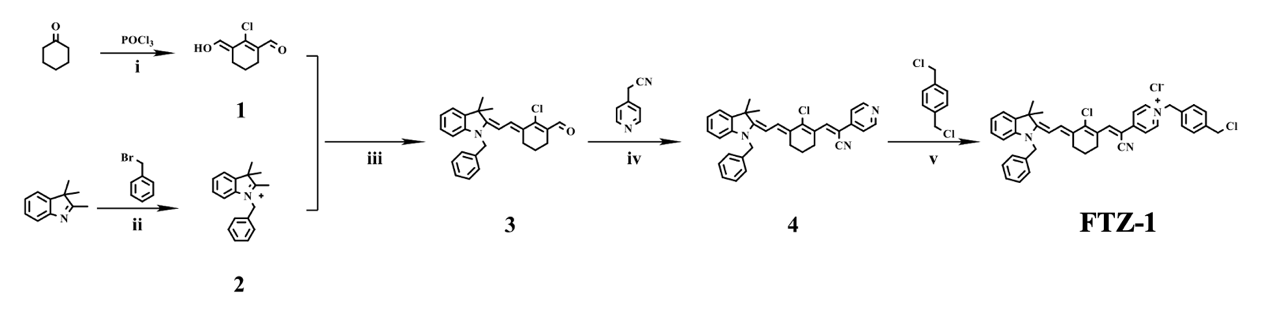


Scheme S1. The synthetic route of FTZ-1. (i) DMF/DCM = (ii) Toluene, 110 °C, 24 hours. (iii) Toluene/acetic acid = 3:1 (v/v), 110 °C, 4 hours. (iv) EtOH, 100 μL piperidine, 78 °C, 4 h. (v) ACN, 85 °C, 17 h.


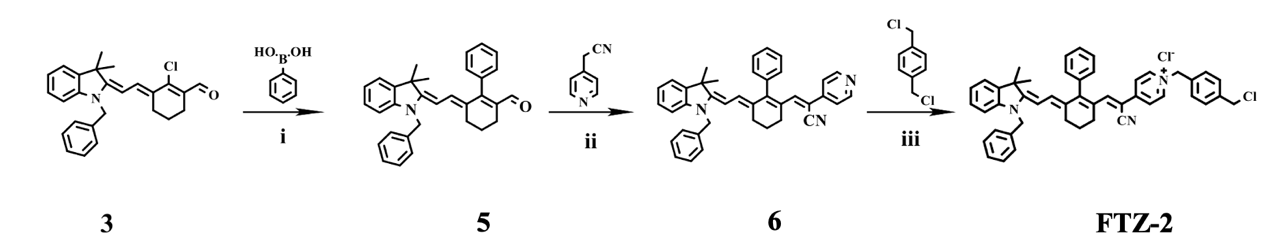


Scheme S2. The synthetic route of FTZ-2. (i) DMF/H_2_O = 5:1 (v/v). (ii) EtOH, 100 μL piperidine, 78 °C, 4 h. (iii) ACN, 85 °C, 17 h.

Table S1. Performance of some mitochondrial-viscosity-responsive fluorescent probes.

| Name | Structure | λ_ex_/λ_em_ (nm) | PCC | Application | Reference |
| --- | --- | --- | --- | --- | --- |
| HTC | 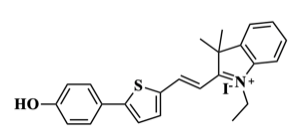 | 520/627 | 0.91 | cell, mice | ^[1]^ |
| Hcy-BA | 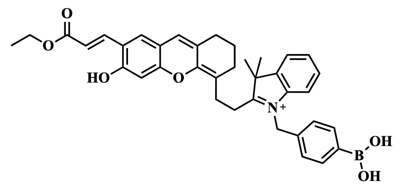 | 640/720 | 0.91 | cell, mice | ^[2]^ |
| Mito-RP | 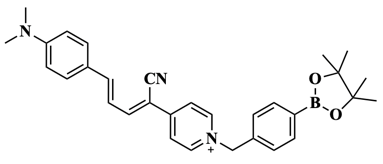 | 600/700 | 0.82 | cell | ^[3]^ |
| MQP-Boc | 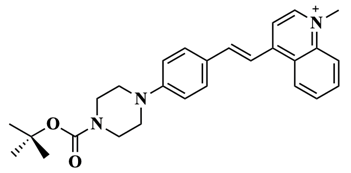 | 510/667 | 0.93 | cell, mice | ^[4]^ |
| TPB | 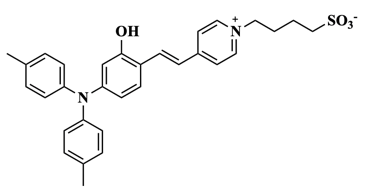 | 485/623 | 0.95 | cell, mice | ^[5]^ |
| JZ-2 | 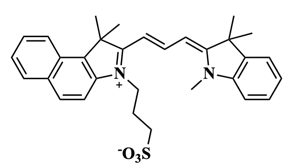 | 560/580 | 0.85 | cell, mice | ^[6]^ |
| MMN | 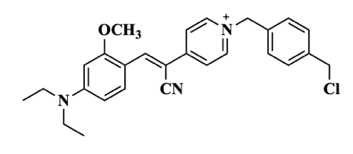 | 530/585 | 0.85 | cell, mice | ^[7]^ |
| WD-2 | 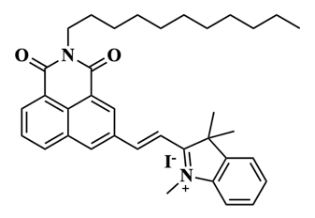 | 500/620 | 0.90 | cell, zebrafish | ^[8]^ |
| NI-QM-OH | 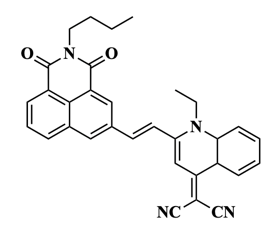 | 500/620 | 0.92 | cell, zebrafish | ^[9]^ |
| YLS | 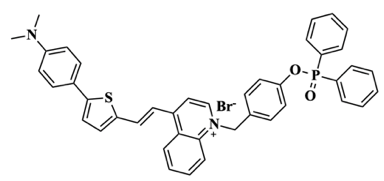 | 590/760 | 0.89 | cell, mice | ^[10]^ |
| HCPVP | 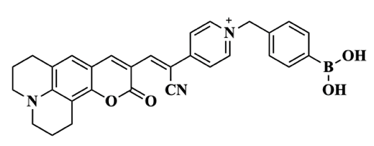 | 600/678 | 0.89 | cell, zebrafish, mice | ^[11]^ |
| This work | 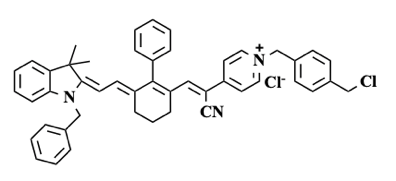 | 780/820 | 0.90 | cell, mice | ^This work^ |

Refence

[1] X. Wang, X. Zhou, Z. Zhang, L. Shen, X. Yan, H. Xu, C. Redshaw, Q. L. Zhang, *J. Mater. Chem. B* **2025**, *13*, 3677-3684.

[2] X. Wang, Y. Chen, C. Liu, R. Sa, X. Hu, J. Chen, N. Fu, G. Wang, *Anal. Chem.* **2025**, *97*, 10244-10251.

[3] Y. Liu, W. Song, W. Dong, X. Gong, C. Dong, J. Zhao, R. Wang, S. Song, S. Shuang, *Talanta* **2025**, *292*, 127909.

[4] S. Zhang, S. Gong, J. Hong, J. Zhang, G. Feng, *Anal. Chem.* **2025**, *97*, 2318-2325.

[5] F. Hu, F. Zhu, L. Yang, X. Wang, S. Zhang, *Sensor. Actuat. B-Chem.* **2025**, *435*, 137644.

[6] J. Zhou, Y. Wang, L. Shi, Y. Liu, X. Zhou, J. Li, H. Ma, J. Zhou, *Anal. Chem.* **2025**, *97*, 9763-9770.

[7] P. Lei, P. Wei, C. Dong, S. Shuang, M. Li, *Sensor. Actuat. B-Chem.* **2025**, *434*, 137614.

[8] D. Wei, Y. Dai, X. Yan, D. Lan, J. Liao, Z. Qin, N. Fu, *ACS Sens.* **2025**, *10*, 2542-2553.

[9] Y. Dai, L. Xiao, J. Liao, Z. Han, N. Fu, *ACS Sens*. **2025**, *10*, 2100-2112.

[10] Q. Pang, F. Huo, Y. Yue, C. Yin, *Chinese Chem. Lett.* **2024**, *36*, 110713.

[11] M. Li, P. Lei, S. Shuang, C. Dong, L. Zhang, *Sensor. Actuat. B-Chem.* **2024**, *413*, 135909.

# 5. Supplementary Figures


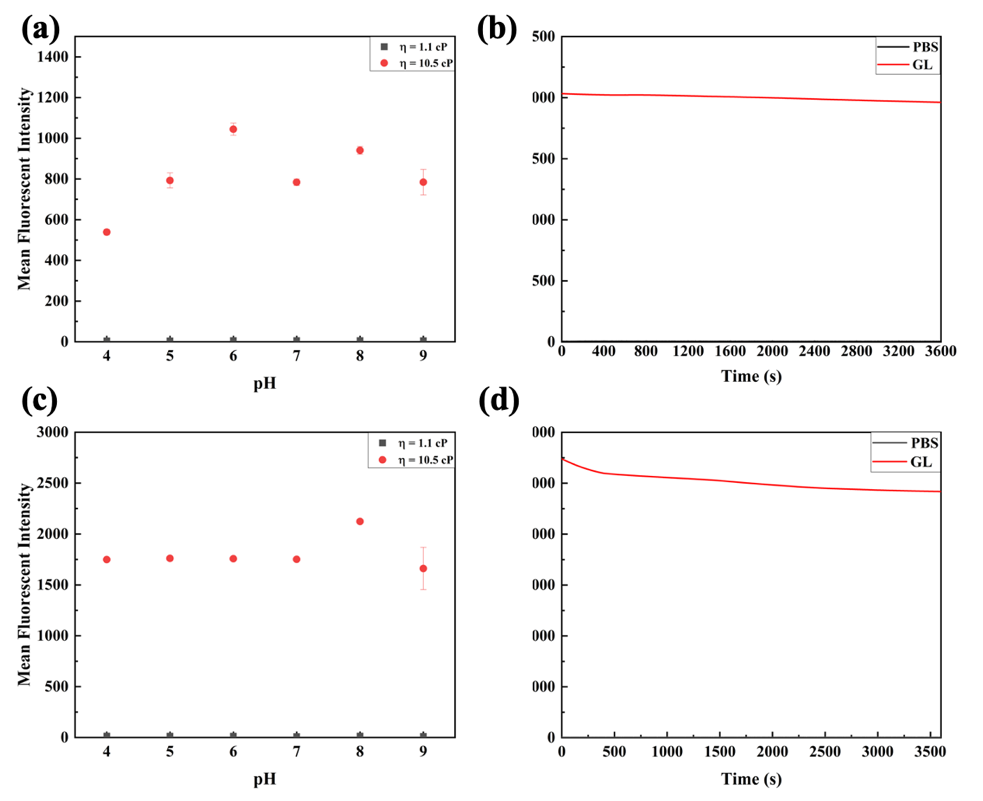


Figure S1. (a) The fluorescence intensity scatter plot of FTZ-1 in different pH solvents. (b) Photostability of FTZ-1 in PBS (black) and GL (red). (c) The fluorescence intensity scatter plot of FTZ-2 in different pH solvents. (d) Photostability of FTZ-2 in PBS (black) and GL (red). λ_ex (FTZ-1)_ = 750 nm, λ_em (FTZ-1)_ = 830 nm. λ_ex (FTZ-2)_ = 780 nm, λ_em (FTZ-2)_ = 820 nm.


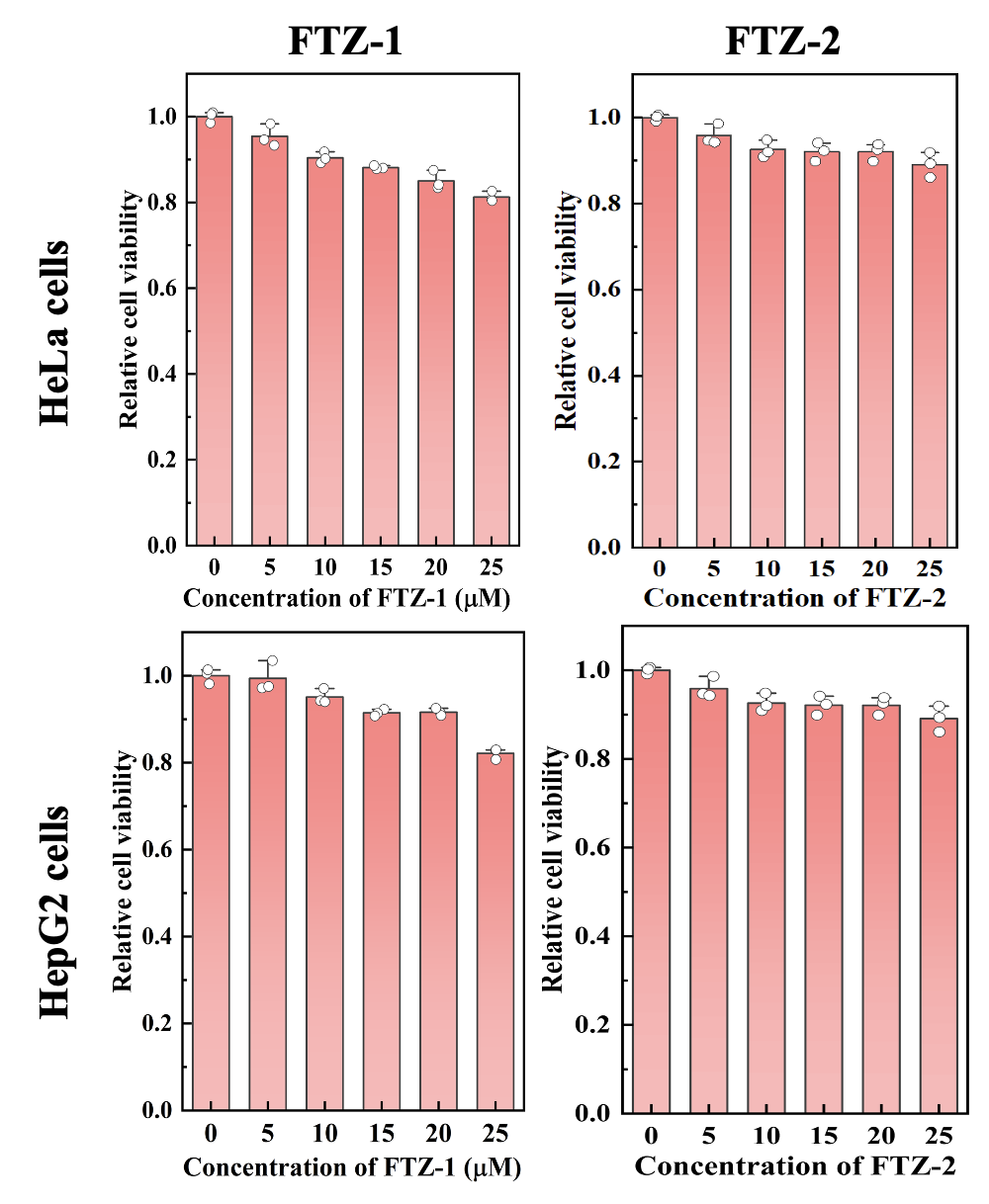


Figure S2. Cytotoxicity of FTZ-1 and FTZ-2 in HeLa cells and HepG2 cells through MTT.


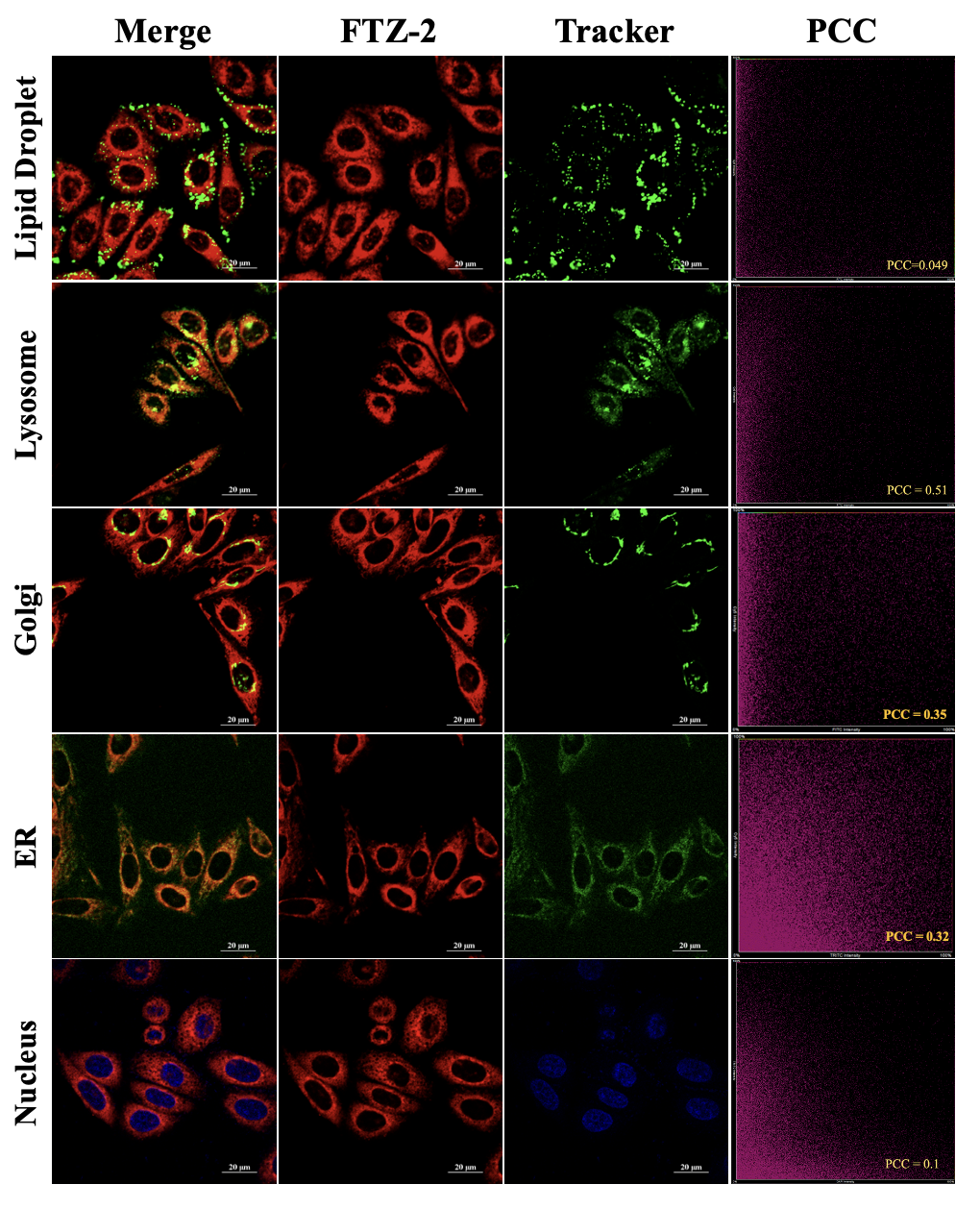


Figure S3. Co-localization experiments of FTZ-2 in HeLa cells. Scale bar: 20 μm.


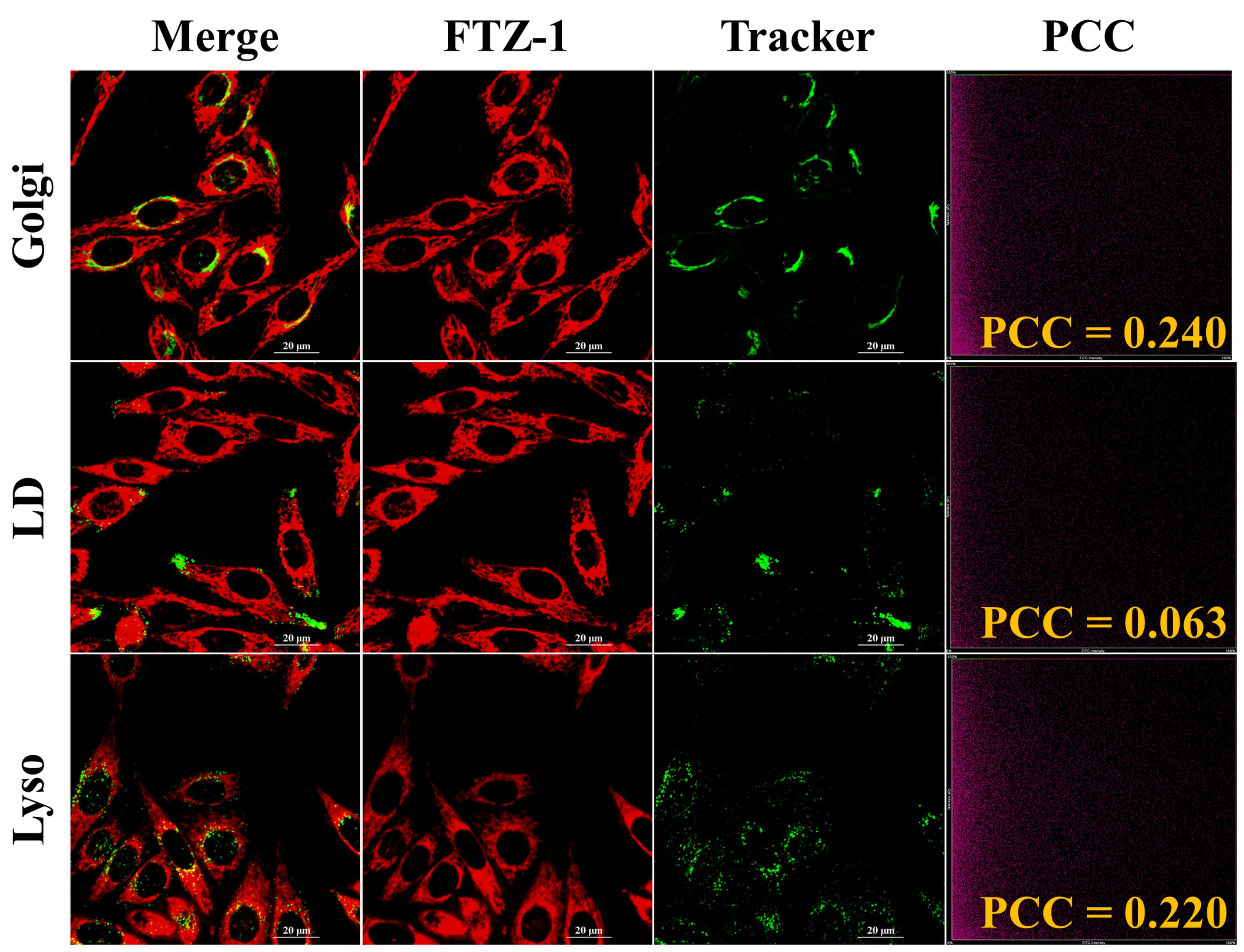


Figure S4. Co-localization experiments of FTZ-1 in HeLa cells. FTZ-1(8 μM, 15 min, λ_ex_ = 640 nm, λ_em_ = 663-738 nm), Golgi-Tracker Green (50 nM, 15 min, λ_ex_ = 488 nm, λ_em_ = 500-550 nm), BODIPY (50 nM, 15 min, λ_ex_ = 488 nm, λ_em_ = 500-550 nm), Lyso-Tracker Green (50 nM, 15 min, λ_ex_ = 488 nm, λ_em_ = 500-550 nm). Scale bar: 20 μm.


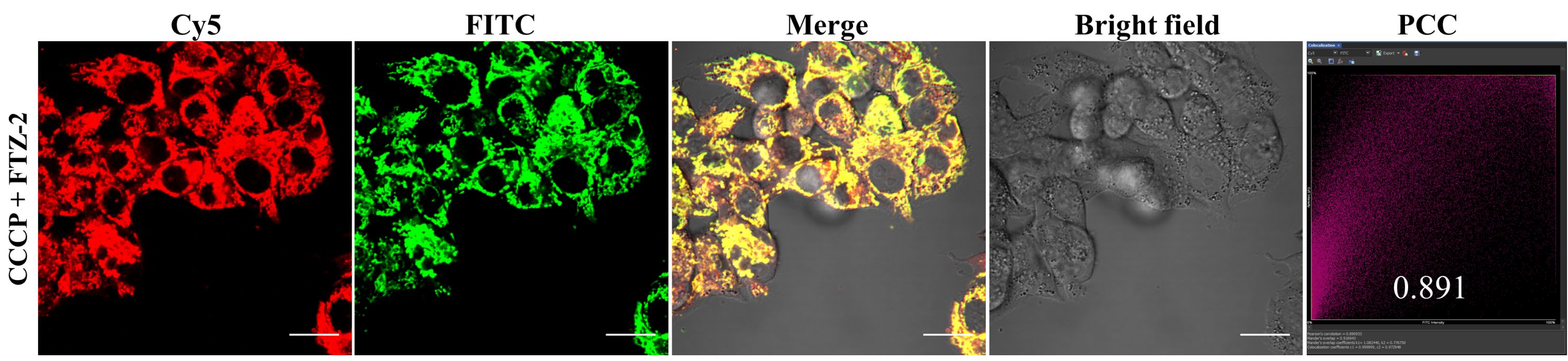


Figure S5. Co-localization imaging of HepG2 cells after mitochondrial depolarization induced by CCCP (20 μM, 2 h) in the presence of FTZ-2. Scale bar: 20 μm.


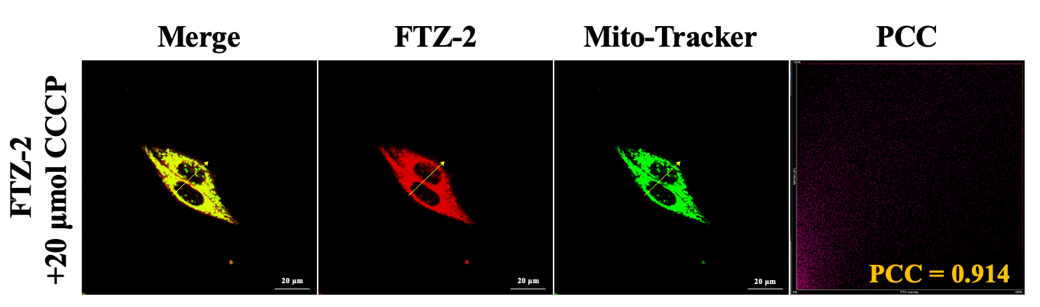


Figure S6. Co-localization imaging of HeLa cells after mitochondrial depolarization induced by CCCP (20 μM, 2 h) in the presence of FTZ-2. Scale bar: 20 μm.


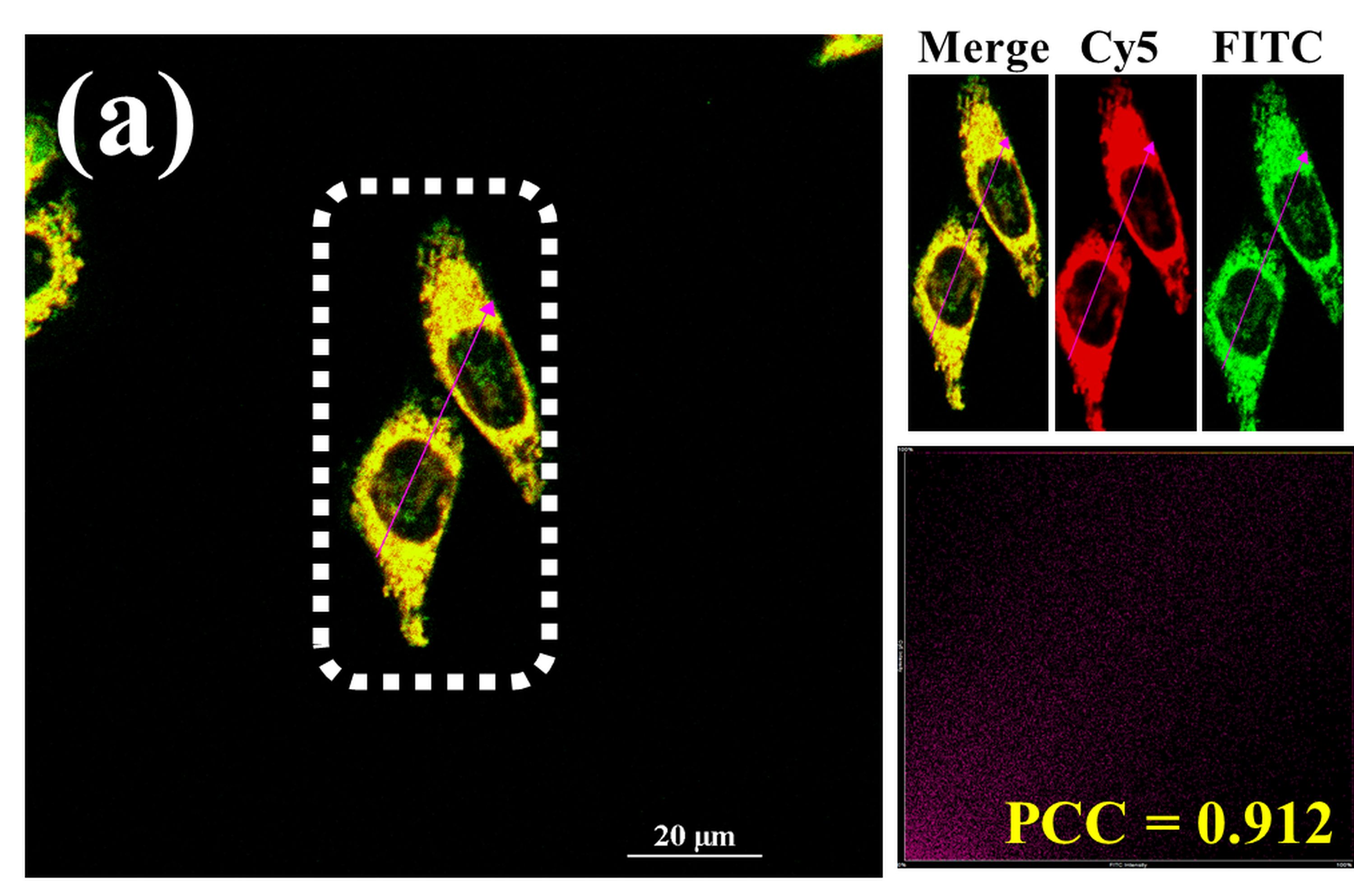


Figure S7. Co-localization imaging of HeLa cells in the presence of FTZ-1 and Mito-Tracker Green. Scale bar: 20 μm.


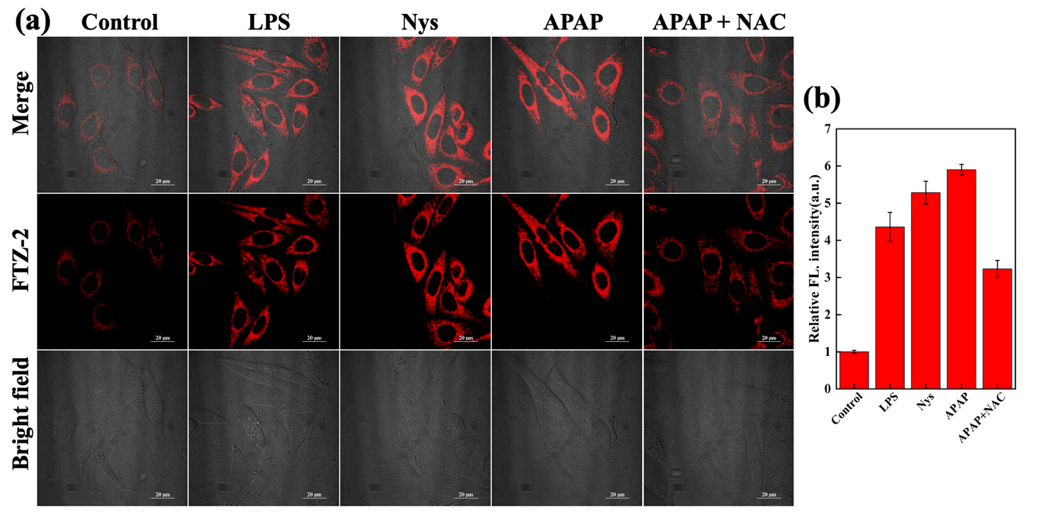


Figure S8. (a) Cellular fluorescence imaging of FTZ-2 in HeLa cells under oxidative stress. (b) Relative fluorescent intensity of each group. λ_ex_ = 640 nm, λ_em_ = 663-738 nm. Scale bar: 20 μm.


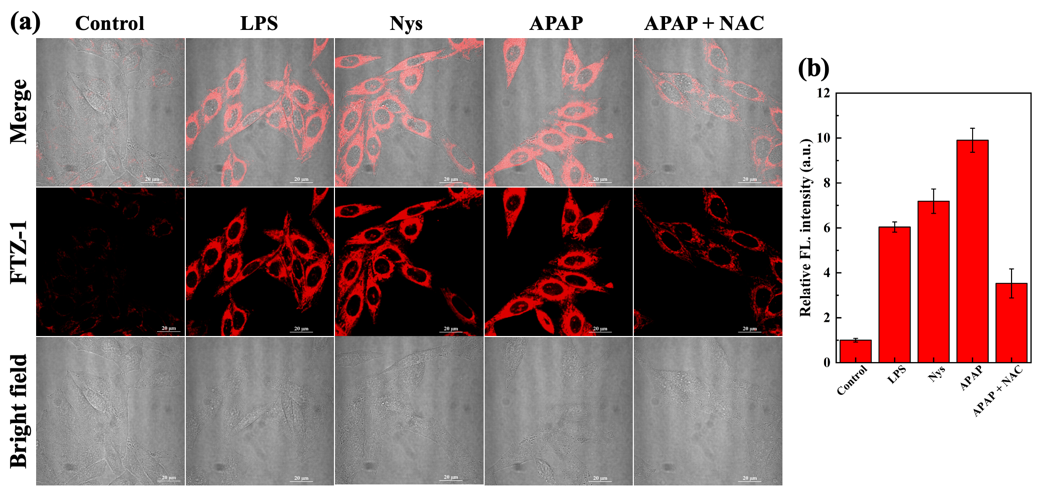


Figure S9. (a) Cellular fluorescence imaging of FTZ-1 in HeLa cells under oxidative stress. (b) Relative fluorescent intensity of each group. λ_ex_ = 640 nm, λ_em_ = 663-738 nm. Scale bar: 20 μm.


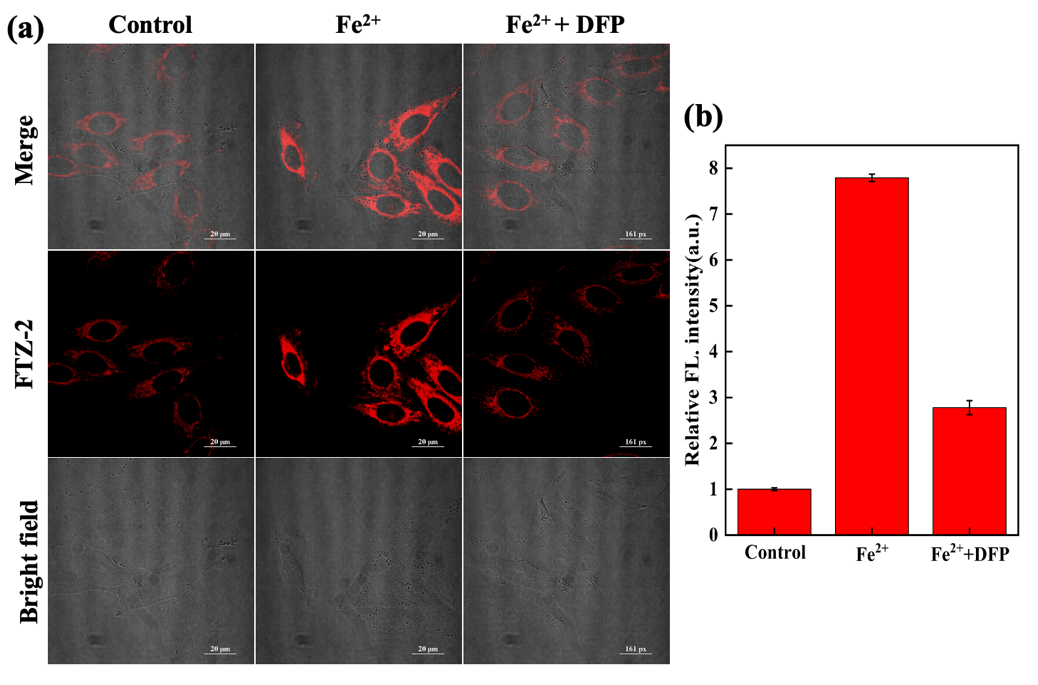


Figure S10. (a) Exogenous ferroptosis imaging of Hela cells. (b) Relative fluorescent intensity of each group. λ_ex_ = 640 nm, λ_em_ = 663-738 nm, Scale bar: 20 μm.


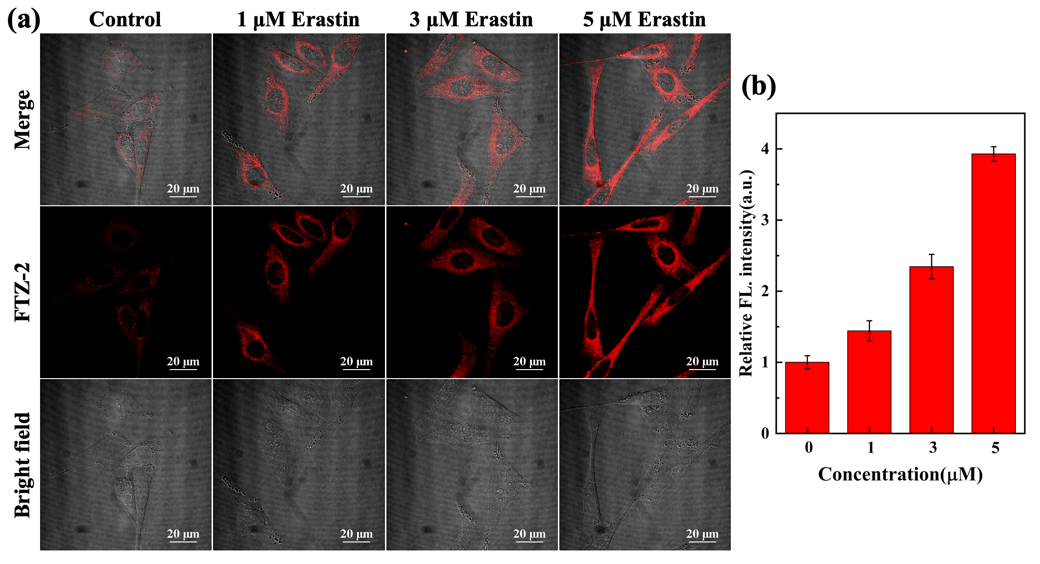


Figure S11. (a) Cell imaging of HeLa cells induced by different concentration of Erastin. (b) Relative fluorescent intensity of each group. λ_ex_ = 640 nm, λ_em_ = 663-738 nm, Scale bar: 20 μm.


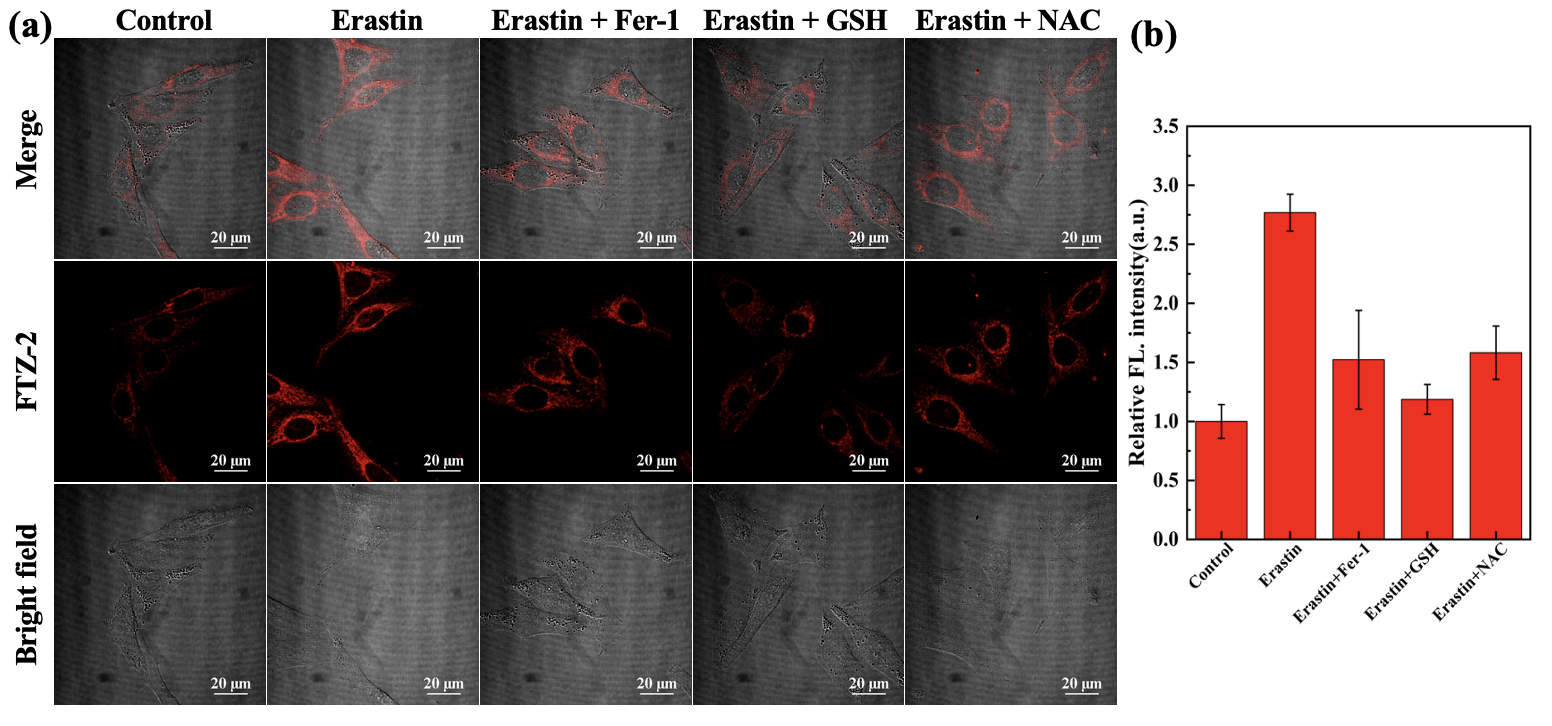


Figure S12. (a) Fluorescence imaging of FTZ-2 in Erastin-induced HeLa cells. (b) Relative fluorescent intensity of cells. λ_ex_ = 640 nm, λ_em_ = 663-738 nm. Scale bar: 20 μm.


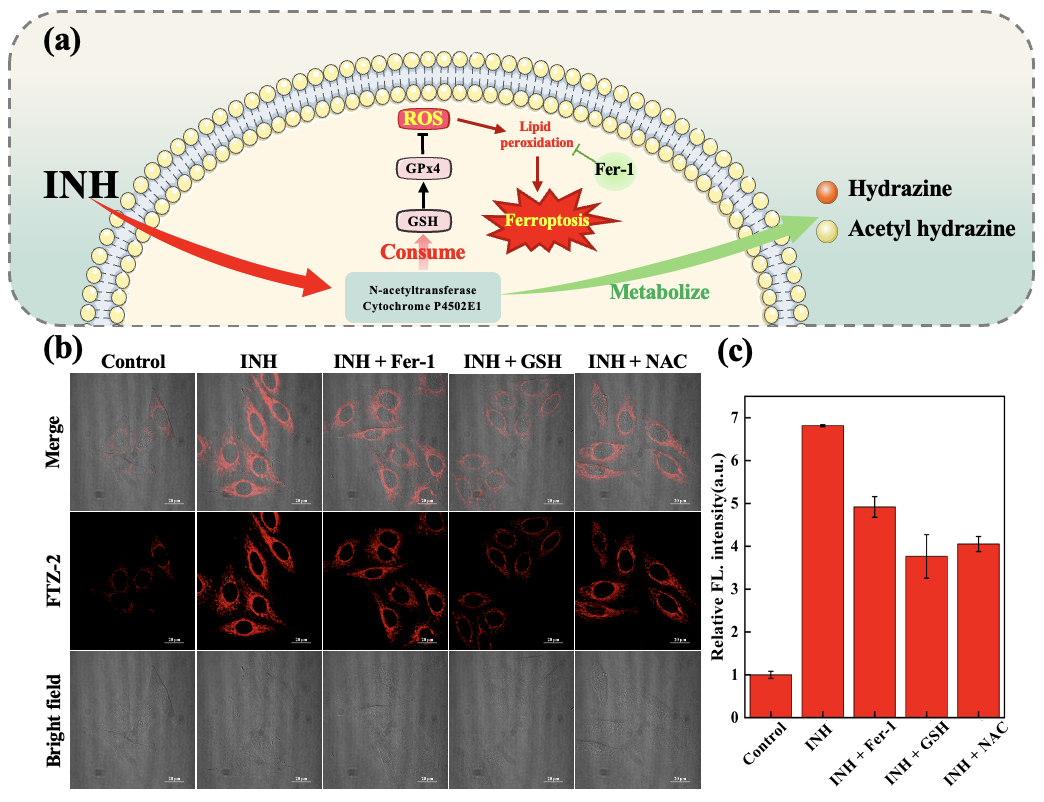


Figure S13. (a) Schematic diagram of INH induced ferroptosis. (b) Fluorescence imaging of FTZ-2 in INH-induced HeLa cells. (c) Relative fluorescent intensity of cells. λ_ex_ = 640 nm, λ_em_ = 663-738 nm. Scale bar: 20 μm.


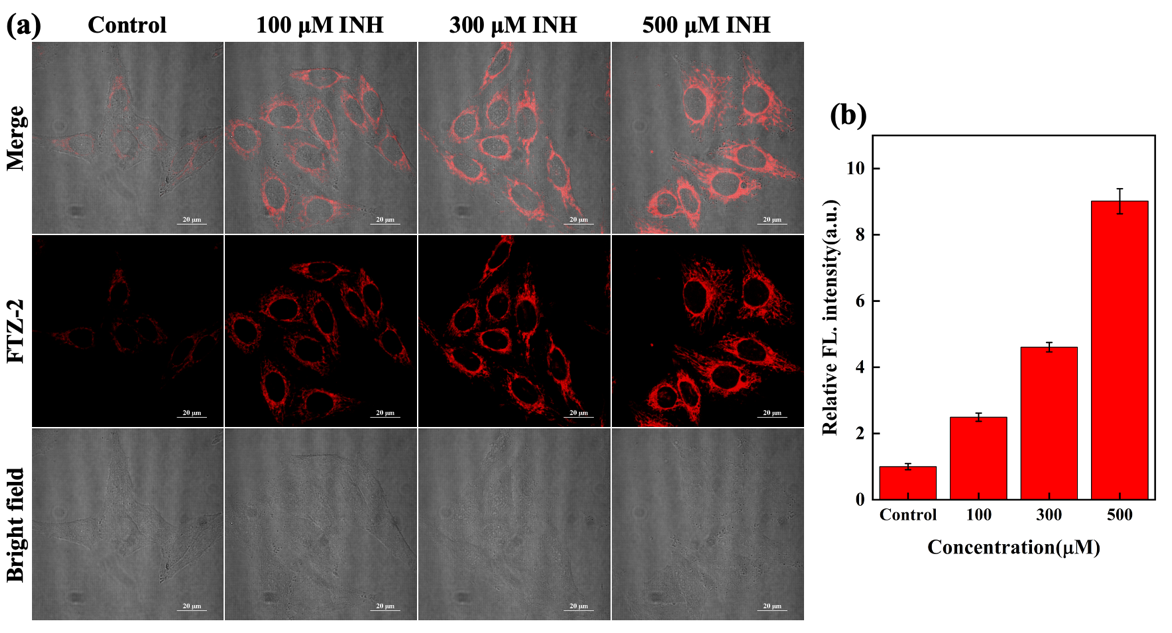


Figure S14. (a) Cell imaging of HeLa cells induced by different concentration of INH. (b) Relative fluorescent intensity of each group. λ_ex_ = 640 nm, λ_em_ = 663-738 nm, Scale bar: 20 μm.


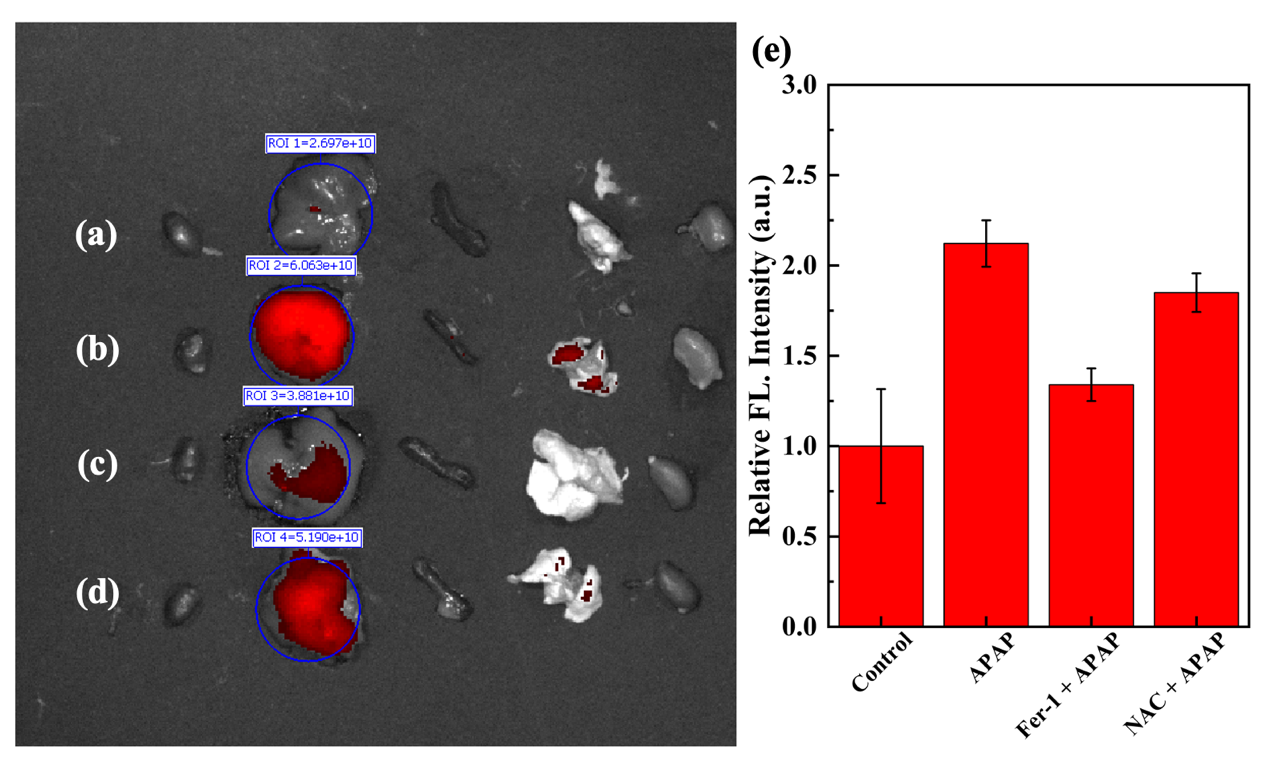


Figure S15. Fluorescence imaging of the heart, liver, spleen, lung, and kidney of each group mice. (a) Control group. (b) APAP group. (c) Fer-1 + APAP group. (d) NAC + APAP group. (e) Relative fluorescent intensity of each four groups. λ_ex_ = 740 nm, λ_em_ = 790 nm.


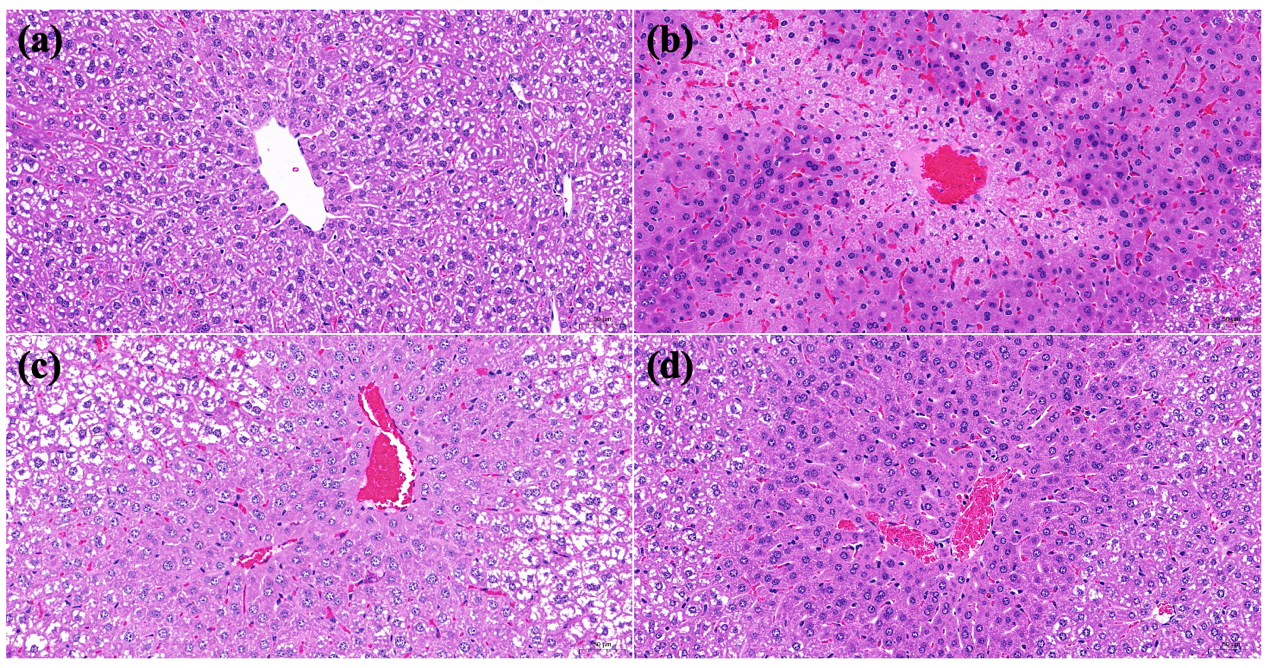


Figure S16. The liver H&E staining of each group mice. (a) Control group. (b) APAP group. (c) Fer-1 + APAP group. (d) NAC + APAP group. Scale bar: 50 μm.


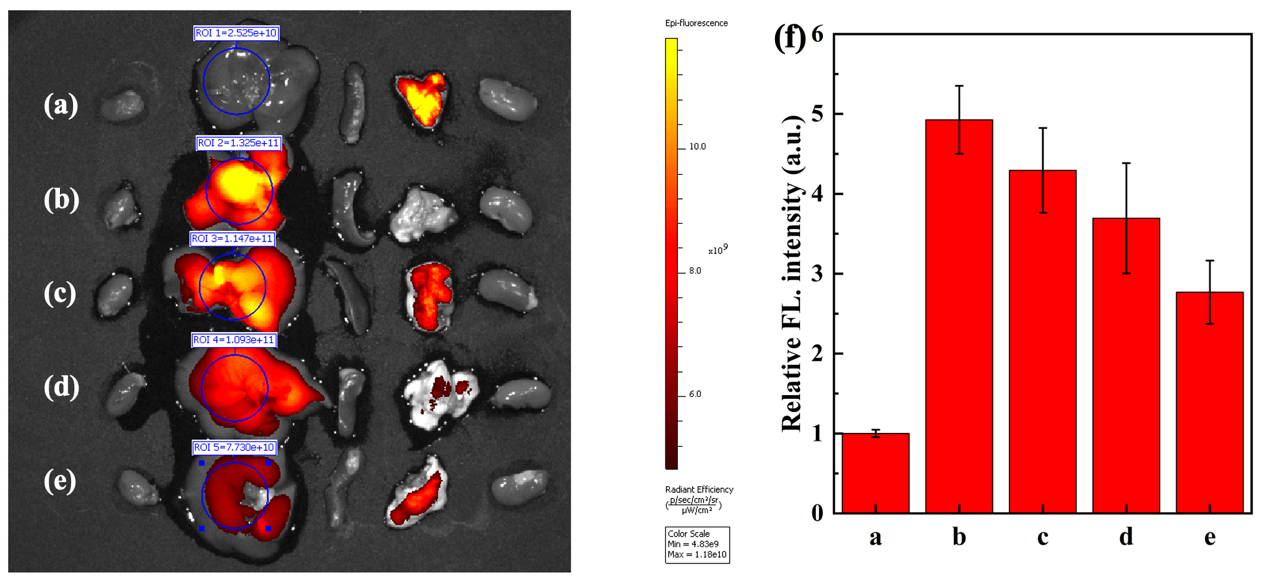


Figure S17. Fluorescence imaging of the heart, liver, spleen, lung, and kidney of each group mice. (a) Control group. (b) APAP + erastin group. (c) Erastin group. (d) Fer-1 + APAP +erastin group. (e) Fer-1 + erastin group. (e) Relative fluorescent intensity of each group. λ_ex_ = 740 nm, λ_em_ = 790 nm.


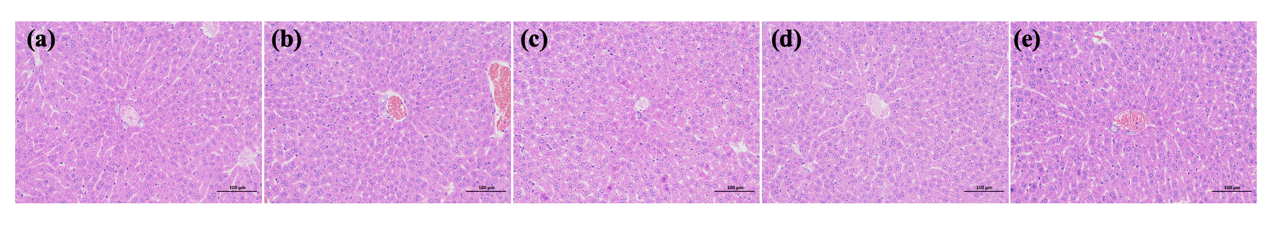


Figure S18. H&E staining of the heart, liver, spleen, lung, and kidney of each group mice. (a) Control group. (b) APAP + erastin group. (c) Erastin group. (d) Fer-1 + APAP + erastin group (e) Fer-1 + erastin group.

# 6. ^1^H NMR, ^13^C NMR and HRMS Spectra


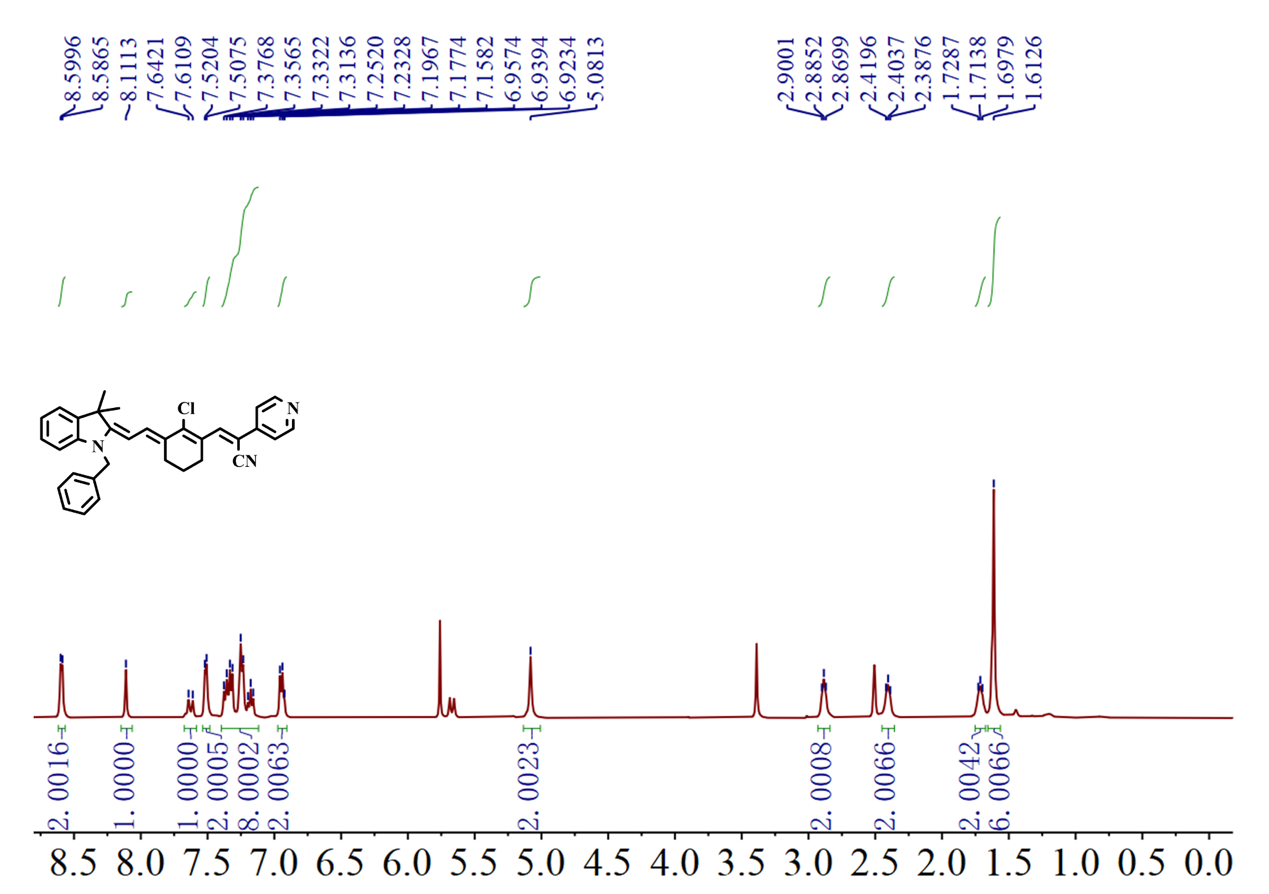


Figure S19. ^1^H-NMR spectrum of compound 4 in DMSO-d_6_ (400 MHz).


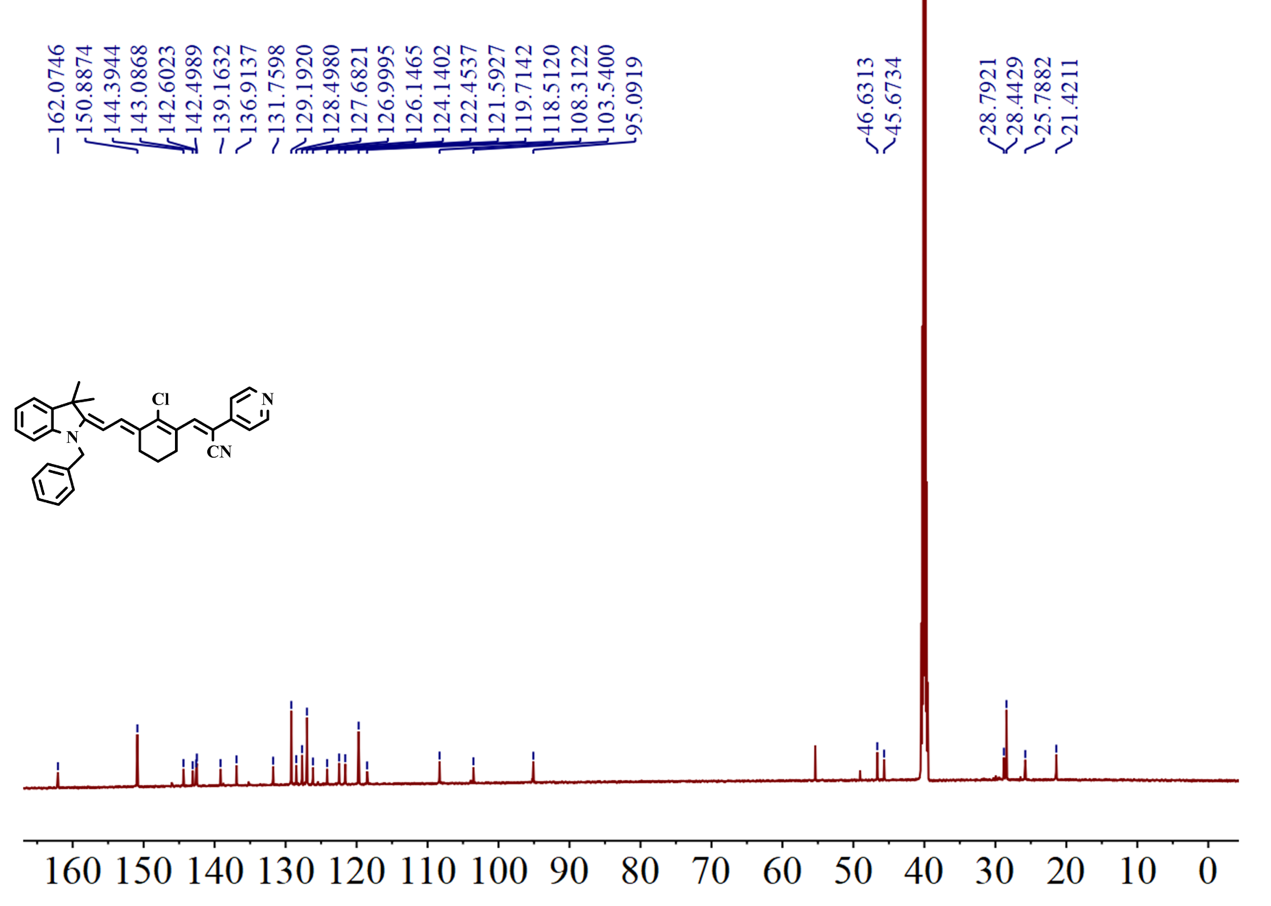


Figure S20. ^13^C-NMR spectrum of compound 4 in DMSO-d_6_ (150 MHz).


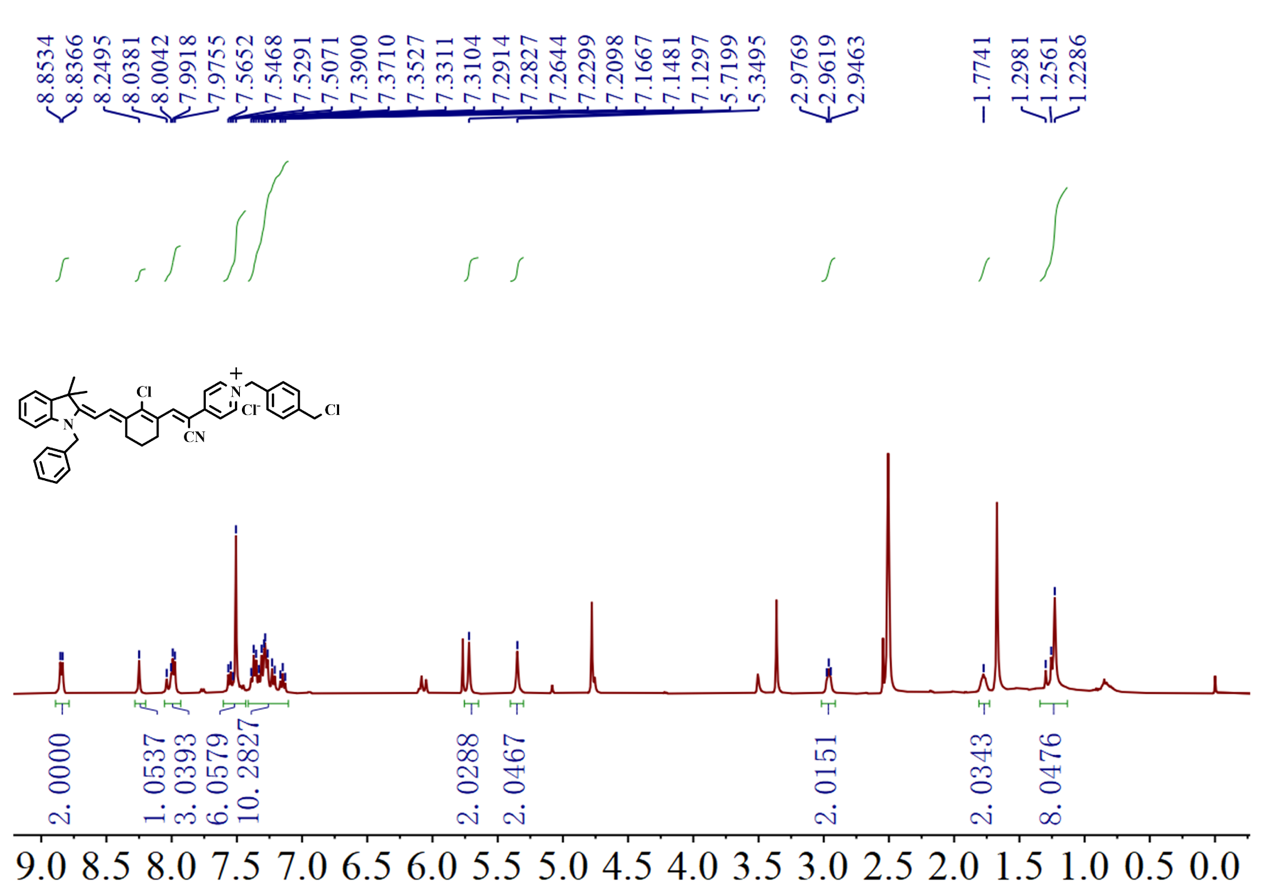


Figure S21. ^1^H-NMR spectrum of FTZ-1 in DMSO-d_6_ (400 MHz).


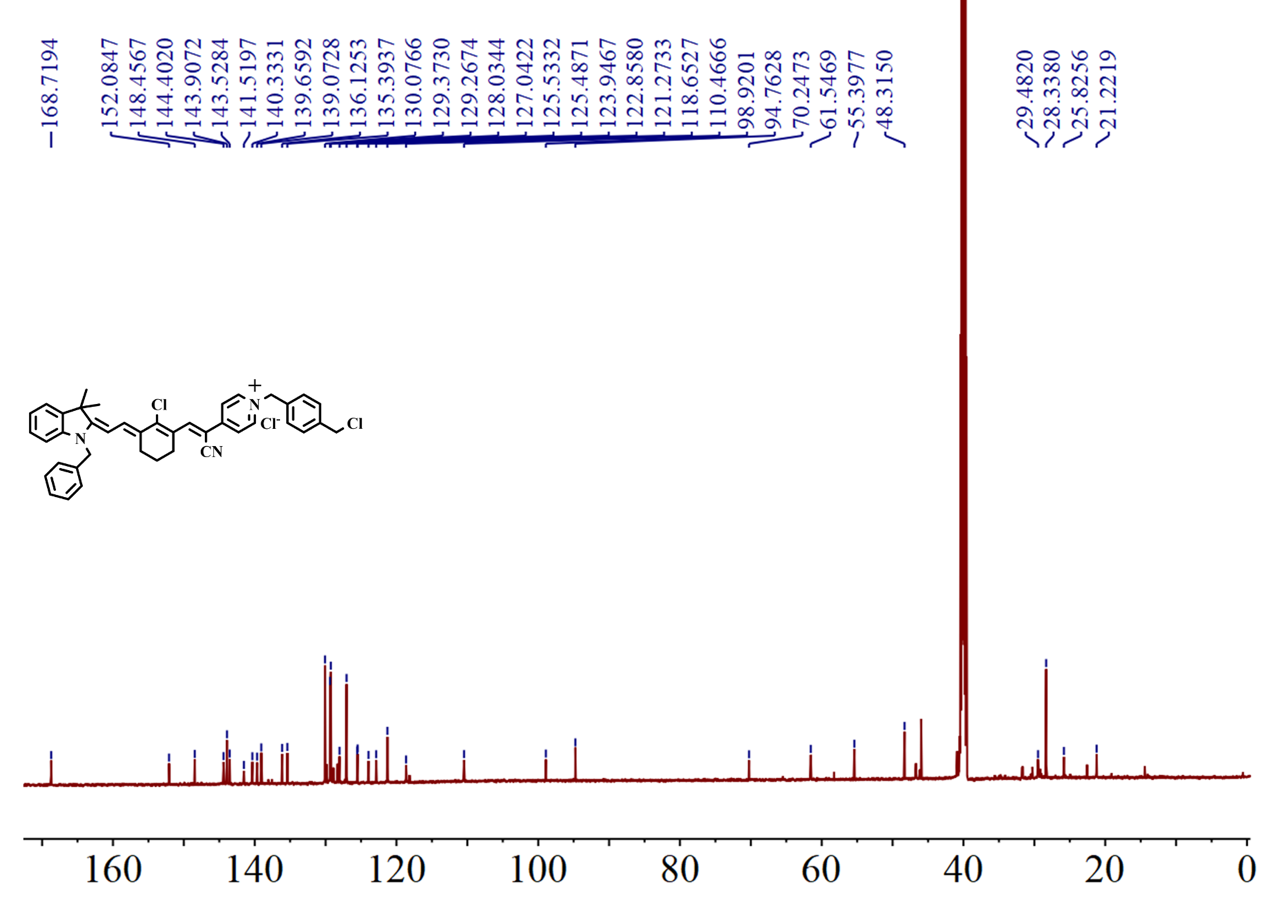


Figure S22. ^13^C-NMR spectrum of FTZ-1 in DMSO-d_6_ (150 MHz).


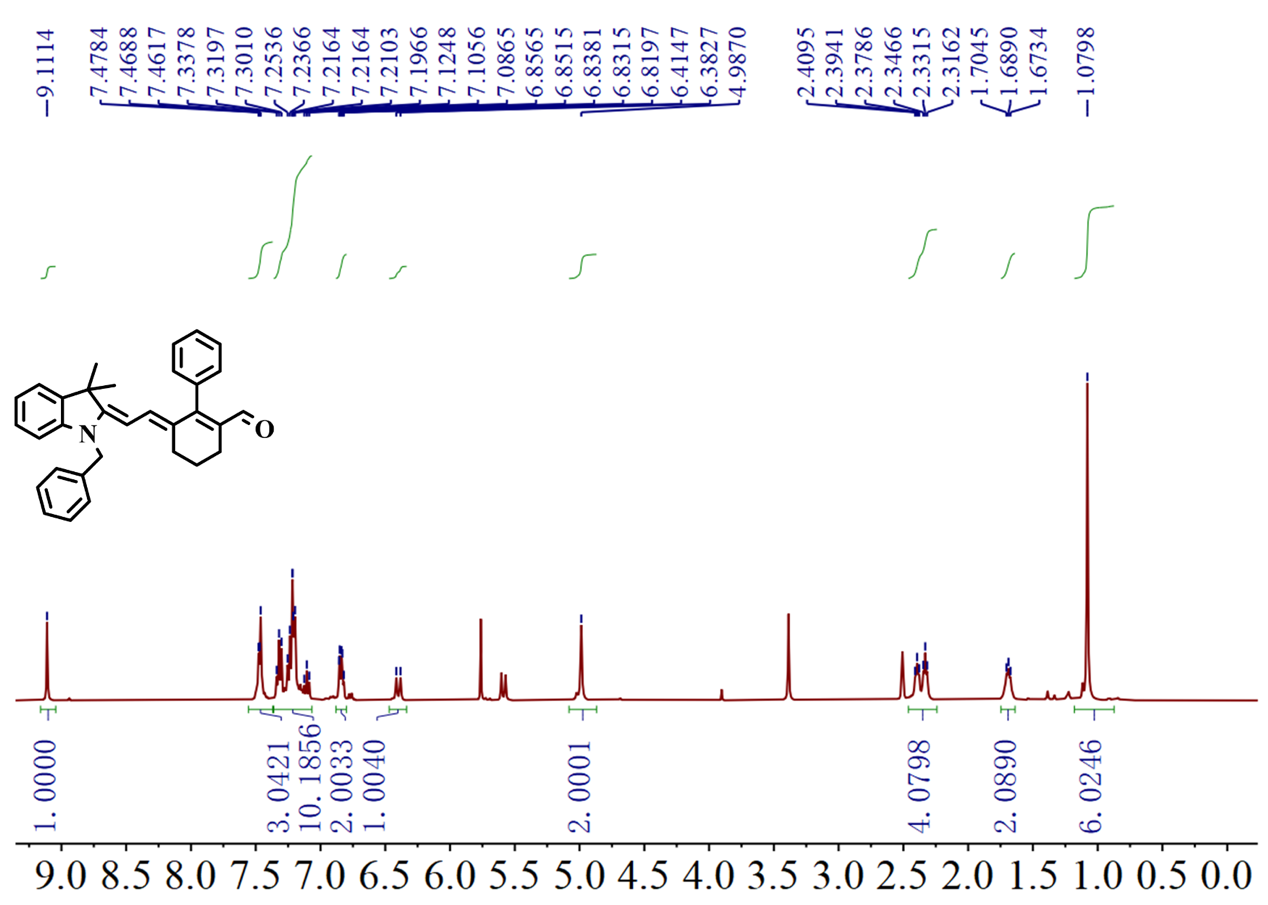


Figure S23. ^1^H-NMR spectrum of compound 5 in DMSO-d_6_ (400 MHz).


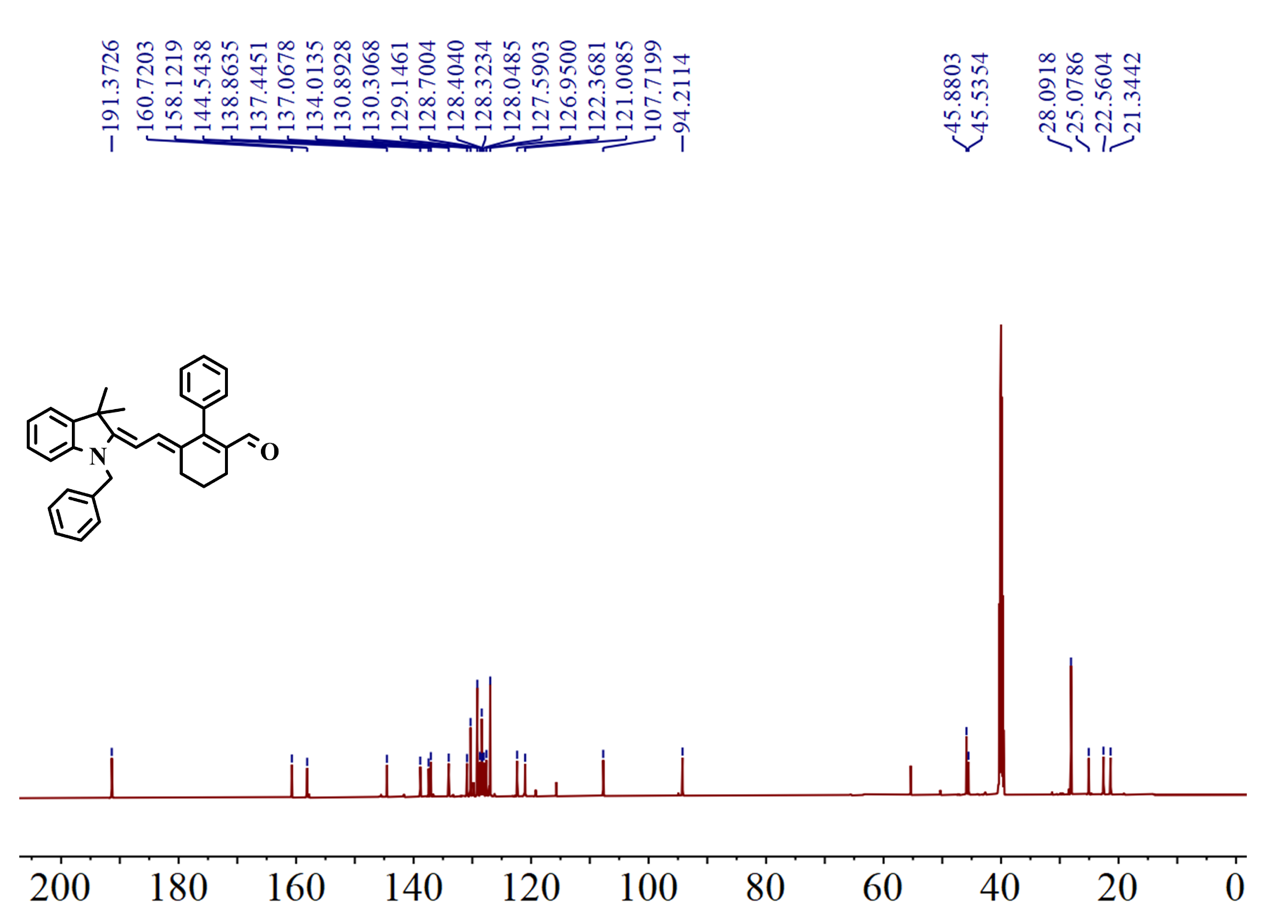


Figure S24. ^13^C-NMR spectrum of compound 5 in DMSO-d_6_ (150 MHz).


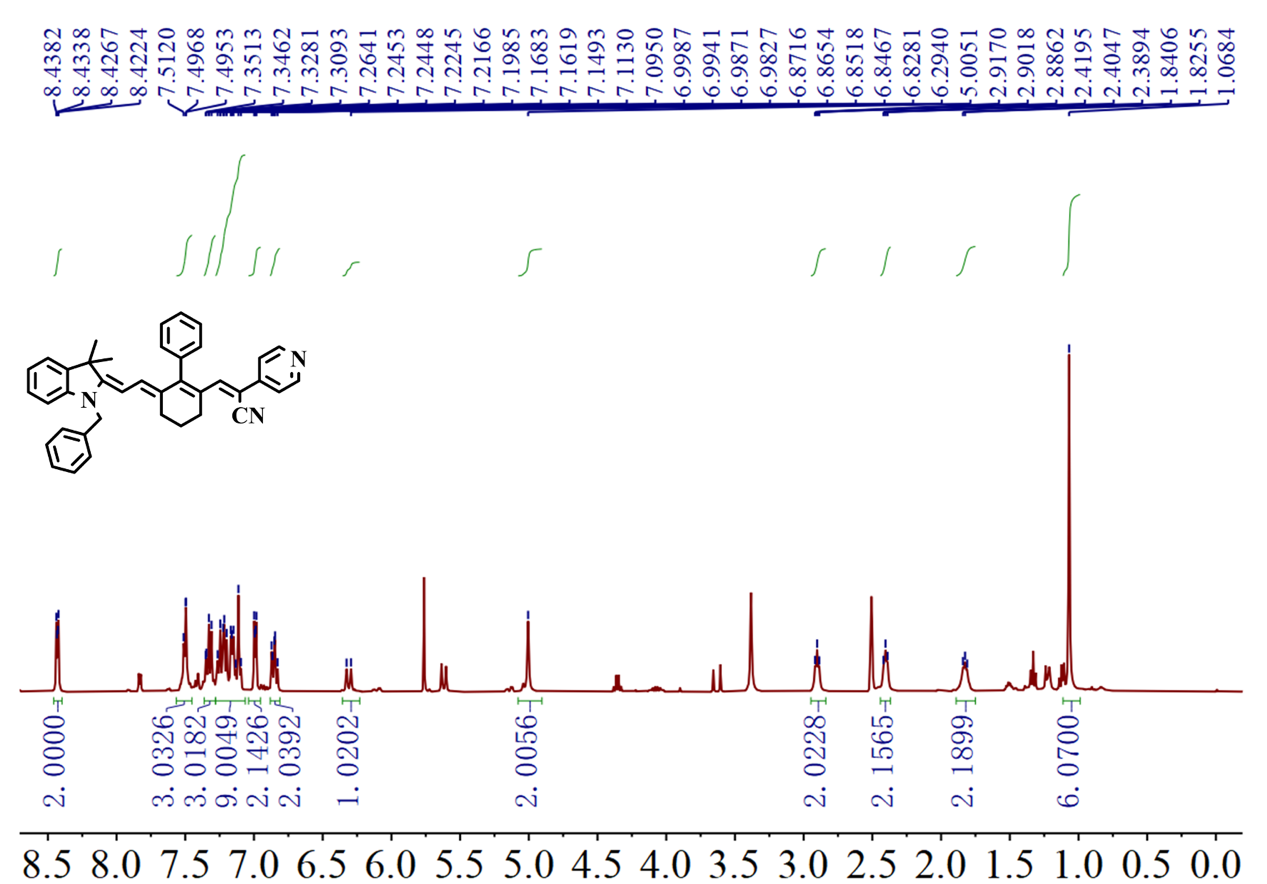


Figure S25 ^1^H-NMR spectrum of compound 6 in DMSO-d_6_ (400 MHz).


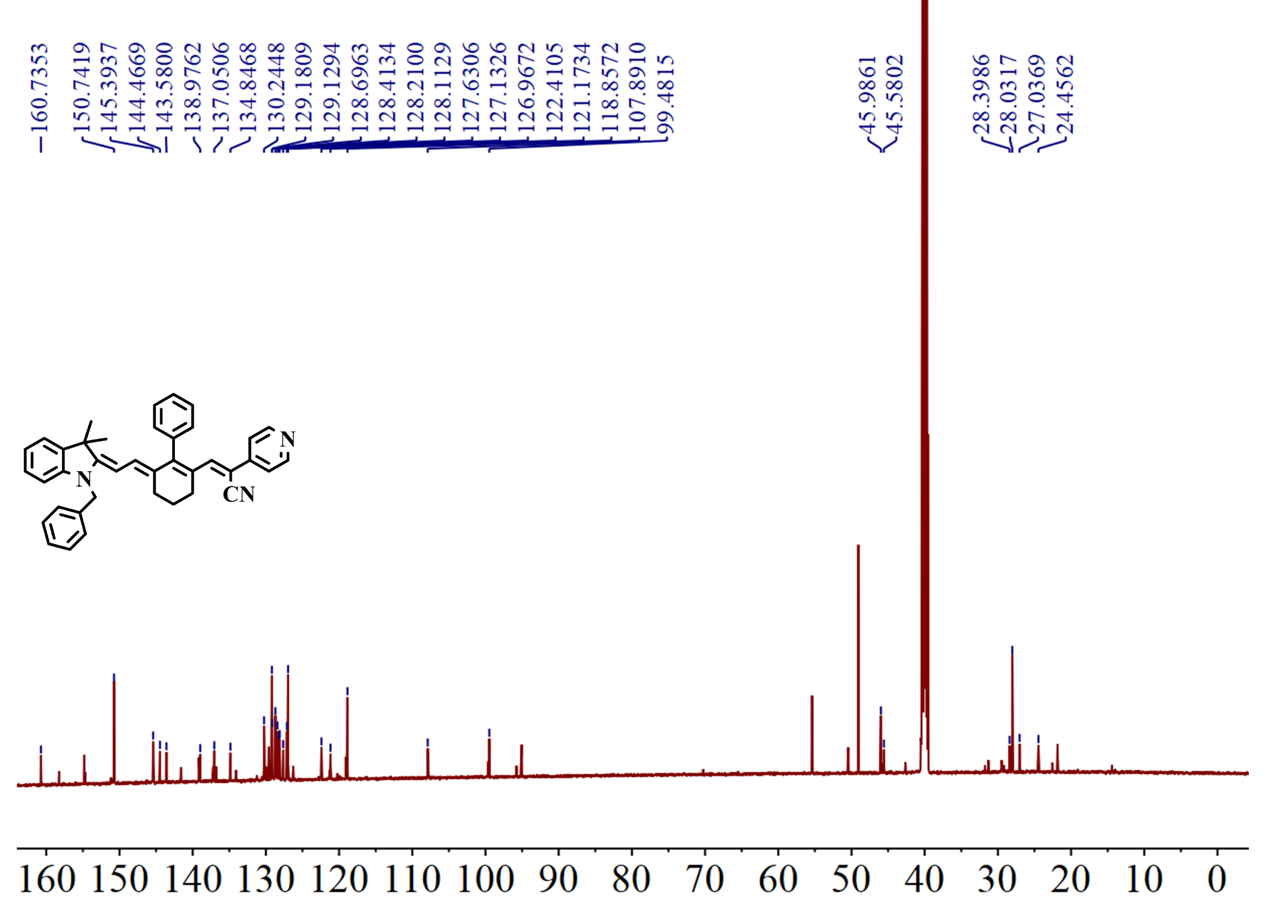


Figure S26. ^13^C-NMR spectrum of compound 6 in DMSO-d_6_ (150 MHz).


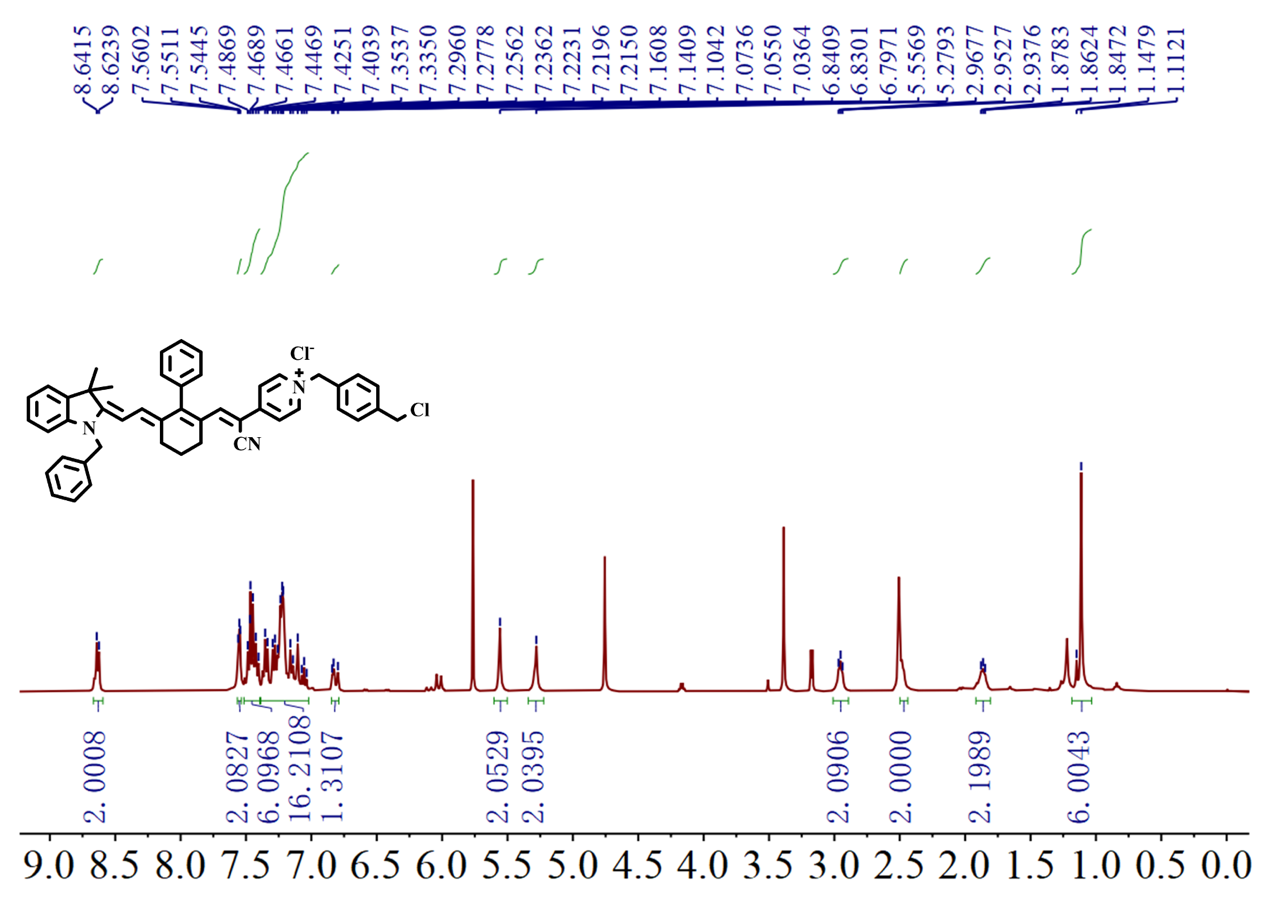


Figure S27. ^1^H-NMR spectrum of FTZ-2 in DMSO-d_6_ (400 MHz).


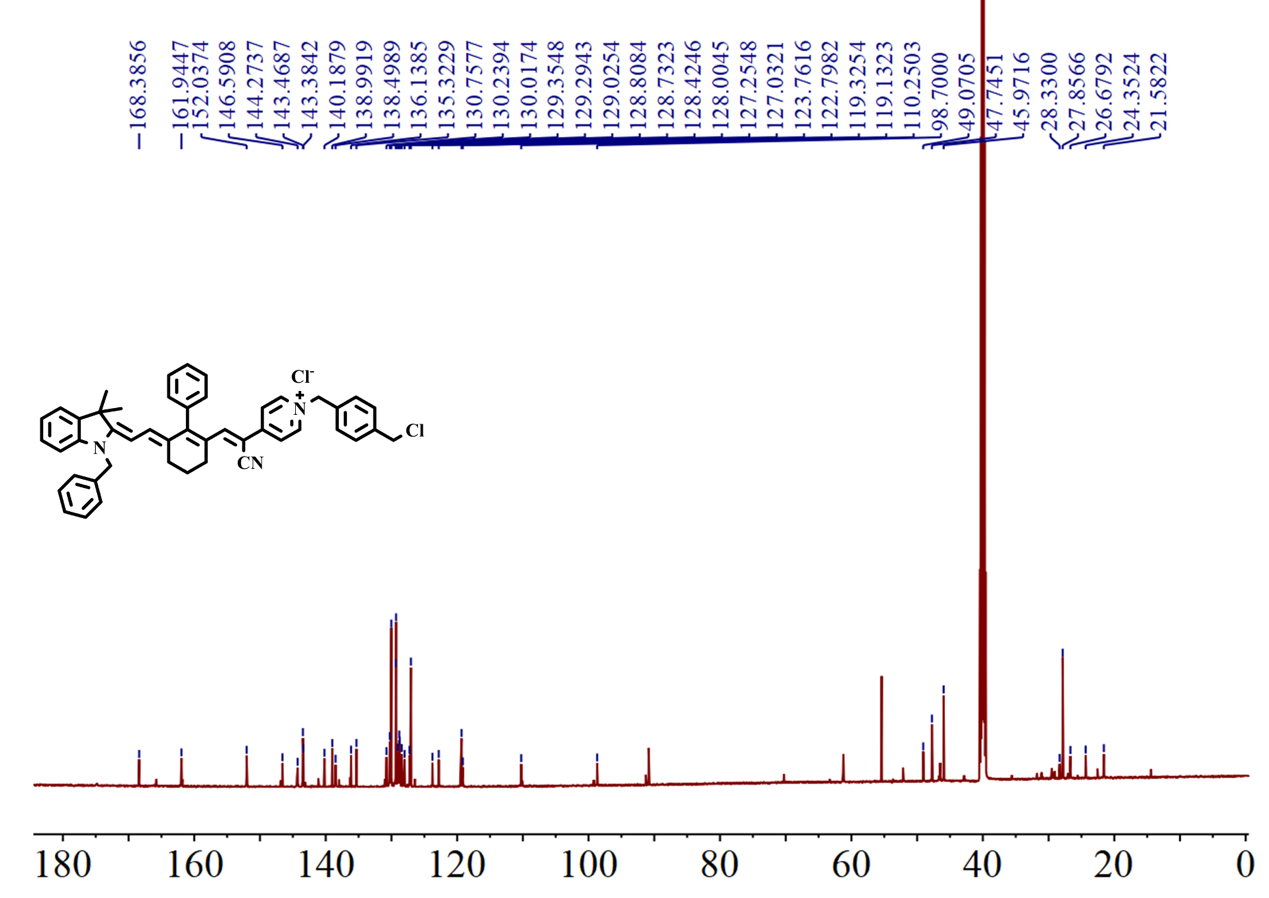


Figure S28. ^13^C-NMR spectrum of FTZ-2 in DMSO-d_6_ (150 MHz).


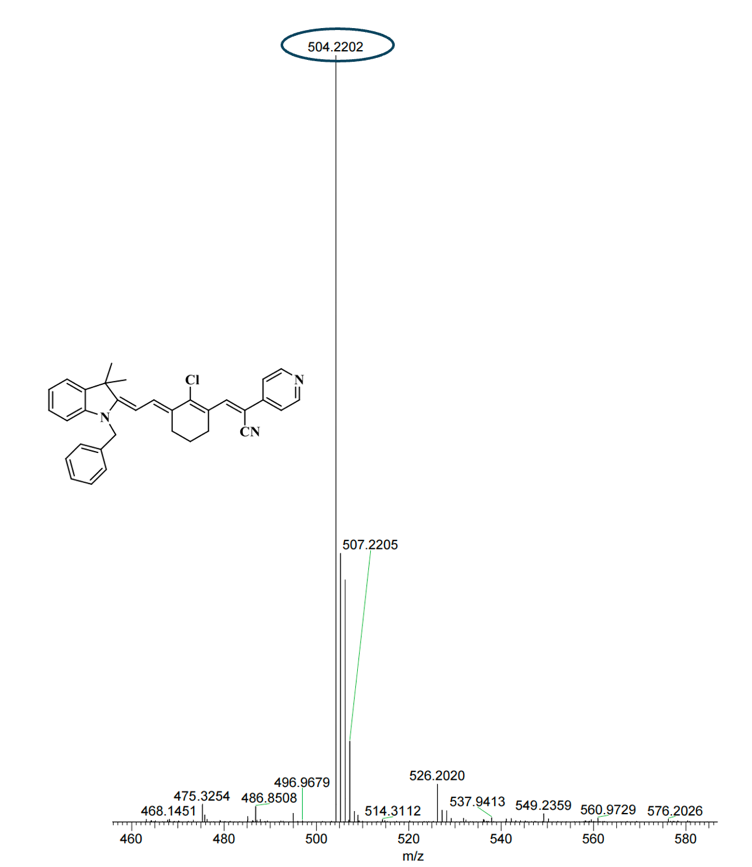


Figure S29. HRMS spectrum of compound 4.


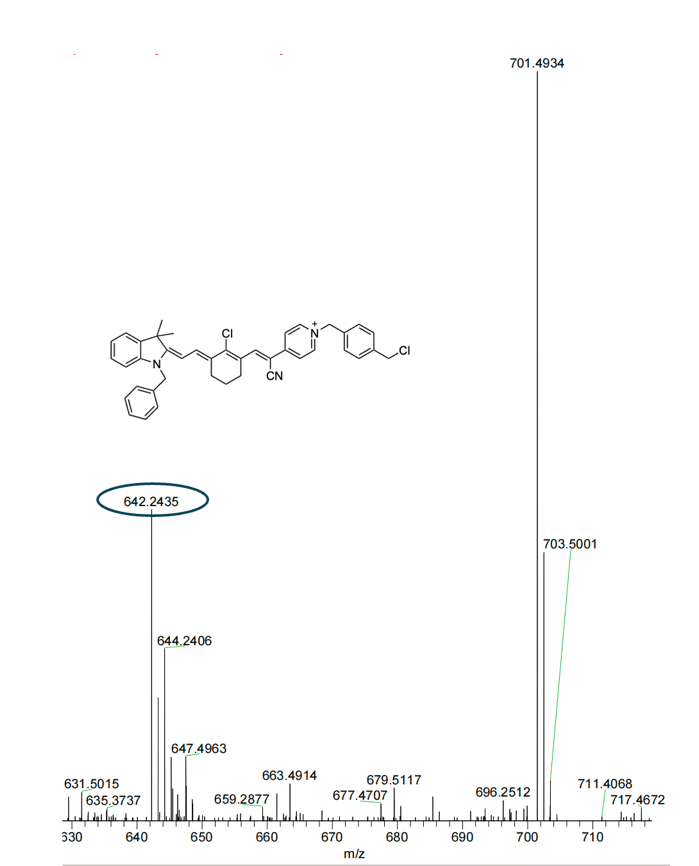


Figure S30. HRMS spectrum of FTZ-1.


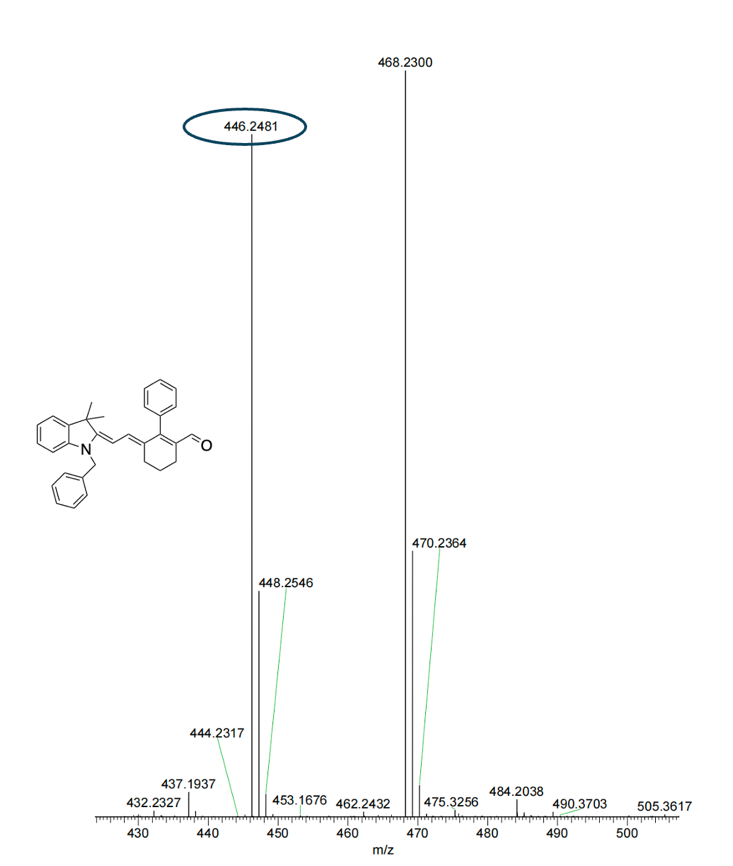


Figure S31. HRMS spectrum of compound 5.


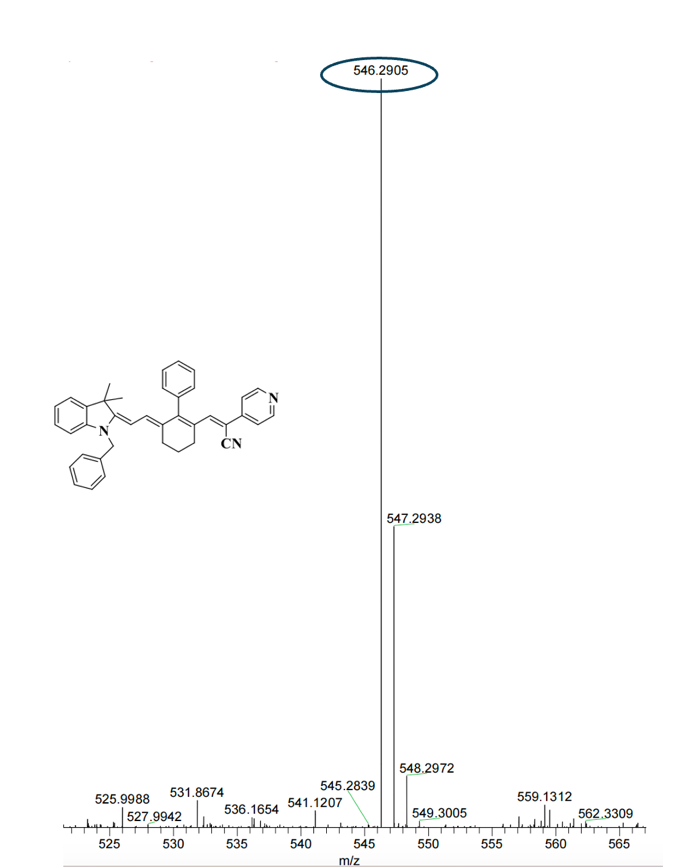


Figure S32. HRMS spectrum of compound 6.


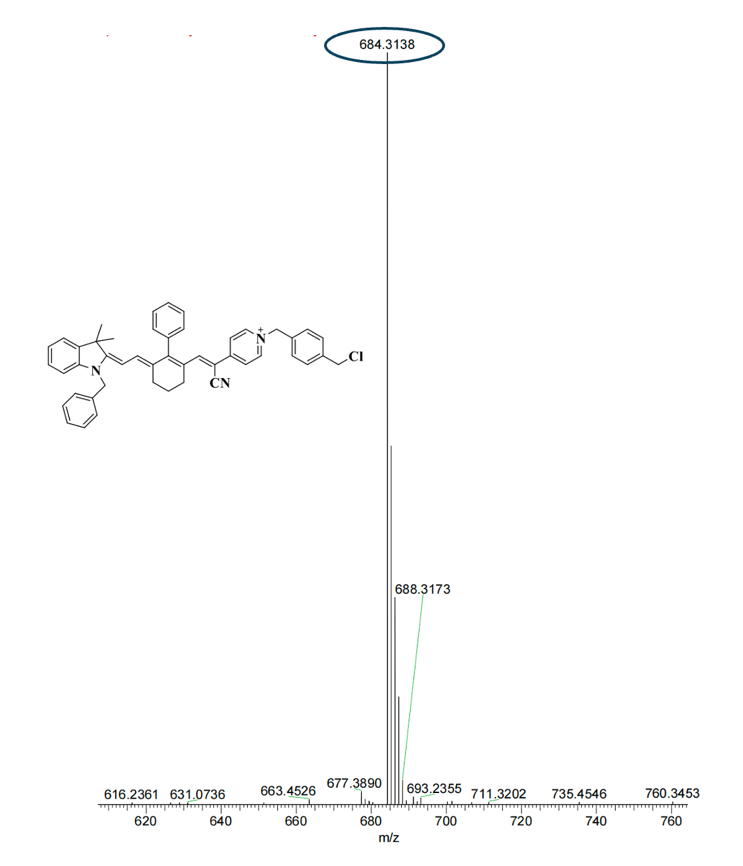


Figure S33. HRMS spectrum of FTZ-2.
